# Improving the response to oxaliplatin by targeting chemotherapy-induced CLDN1 in resistant metastatic colorectal cancer cells

**DOI:** 10.1186/s13578-023-01015-5

**Published:** 2023-04-11

**Authors:** Sara Cherradi, Véronique Garambois, Johanna Marines, Augusto Faria Andrade, Alexandra Fauvre, Olivia Morand, Manon Fargal, Ferial Mancouri, Adeline Ayrolles-Torro, Nadia Vezzo-Vié, Marta Jarlier, Gerald Loussaint, Steve Huvelle, Nicolas Joubert, Thibault Mazard, Céline Gongora, Philippe Pourquier, Florence Boissière-Michot, Maguy Del Rio

**Affiliations:** 1 Institut de Recherche en Cancérologie de Montpellier, INSERM U1194, Université de Montpellier, Institut du Cancer de Montpellier, 208 rue des Apothicaires, Montpellier Cedex 5, F-34298 France; 2 Biometry Department, ICM, Montpellier, France; 3 Department of Medical Oncology, ICM, Montpellier, France; 4 Translational Research Unit, ICM, Montpellier, France; 5 GICC, Team IMT, University of Tours, Tours, 7501, F-37032 France

**Keywords:** Colorectal cancer, CLDN1, ADC, Chemotherapy, Resistance, “One-two punch”, Biomarker

## Abstract

**Background:**

Tumor resistance is a frequent cause of therapy failure and remains a major challenge for the long-term management of colorectal cancer (CRC). The aim of this study was to determine the implication of the tight junctional protein claudin 1 (CLDN1) in the acquired resistance to chemotherapy.

**Methods:**

Immunohistochemistry was used to determine CLDN1 expression in post-chemotherapy liver metastases from 58 CRC patients. The effects of oxaliplatin on membrane CLDN1 expression were evaluated by flow cytometry, immunofluorescence and western blotting experiments in vitro and in vivo. Phosphoproteome analyses, proximity ligation and luciferase reporter assays were used to unravel the mechanism of CLDN1 induction. RNAseq experiments were performed on oxaliplatin-resistant cell lines to investigate the role of CLDN1 in chemoresistance. The “one-two punch” sequential combination of oxaliplatin followed by an anti-CLDN1 antibody-drug conjugate (ADC) was tested in both CRC cell lines and murine models.

**Results:**

We found a significant correlation between CLDN1 expression level and histologic response to chemotherapy, CLDN1 expression being the highest in resistant metastatic residual cells of patients showing minor responses. Moreover, in both murine xenograft model and CRC cell lines, CLDN1 expression was upregulated after exposure to conventional chemotherapies used in CRC treatment. CLDN1 overexpression was, at least in part, functionally related to the activation of the MAPKp38/GSK3β/Wnt/β-catenin pathway. Overexpression of CLDN1 was also observed in oxaliplatin-resistant CRC cell lines and was associated with resistance to apoptosis, suggesting an anti-apoptotic role for CLDN1. Finally, we demonstrated that the sequential treatment with oxaliplatin followed by an anti-CLDN1 ADC displayed a synergistic effect in vitro and in *in vivo.*

**Conclusion:**

Our study identifies CLDN1 as a new biomarker of acquired resistance to chemotherapy in CRC patients and suggests that a “one-two punch” approach targeting chemotherapy-induced CLDN1 expression may represent a therapeutic opportunity to circumvent resistance and to improve the outcome of patients with advanced CRC.

**Supplementary Information:**

The online version contains supplementary material available at 10.1186/s13578-023-01015-5.

## Background

Colorectal cancer (CRC) is the third leading cause of cancer death worldwide [1]. Its prognosis is strongly dependent on the disease stage. When tumors are detected at early stages, surgery remains the primary treatment [2]. However, in most patients, CRC is diagnosed at advanced stages and is usually managed using chemotherapy. Fluoropyrimidine (5-FU), oxaliplatin and irinotecan represent the backbone chemotherapy options for CRC, as single agents or more often in combination (e.g. FOLFOX, FOLFIRI, or FOLFIRINOX) [3]. For patients with metastatic CRC, chemotherapy is often combined with targeted therapy to increase the therapeutic response rate. This includes monoclonal antibodies against EGFR (e.g. cetuximab) or VEGF (bevacizumab), tyrosine kinase inhibitors (e.g. regorafenib, lapatinib), and immune checkpoint blockade agents (e.g. nivolumab, pembrolizumab) according to molecular characteristics of the tumor. However, therapeutic failure occurs mostly due to treatment resistance resulting in decreased survival rates of CRC patients. Resistance to chemotherapy, either intrinsic (primary resistance) or acquired (secondary resistance), defines the tumor cell capacity to escape treatment [4]. Intrinsic resistance is inherent to pre-existing heterogeneity of cancer cells in the tumor while acquired resistance occurs during the course of treatment. Resistance mechanisms in CRC are diverse and include a change in the activity of metabolism enzymes [5], overexpression of efflux pumps [6] alterations of therapeutic target [7], epigenetic changes such as histone acetylation [8], or the presence of specific point mutations in genes involved in drug response. The best example is KRAS mutations that are causally associated with acquired resistance to anti-EGFR therapies [9]. Another example is the aberrant Wnt signaling that plays a crucial role in preventing apoptosis [10] and promoting cancer stem cell features upon 5-FU treatment [11]. Inhibition of this pathway can increase CRC cell sensitivity to oxaliplatin, 5-FU and irinotecan[12].

Accumulating evidences indicate a role of several claudins (tight junction proteins) in chemotherapy resistance. For example, claudin-6 has been implicated in doxorubicin resistance in triple-negative breast cancer [13] and in sorafenib resistance in hepatocellular carcinoma [14]. Claudin-3 and − 4 have been implicated in cisplatin resistance induction in ovarian cancer [15], and claudin-1 in chemotherapy resistance in lung cancer [16]. As claudins are surface proteins, they have attracted interest for tumor targeting by antibodies [17]. For instance, a phase II clinical trial demonstrated that the combination of the anti-claudin-18.2 antibody IMAB362 with chemotherapy significantly prolongs survival of patients with advanced gastric cancer [18].

We previously developed a monoclonal antibody (mAb) that targets the membrane form of claudin-1 (CLDN1) and showed that it could inhibit the growth of colon cancer cells expressing CLDN1, suggesting that CLDN1 targeting could be of clinical benefit for a subset of CRC patients [19]. In the present study we provide further experimental data suggesting a role of CLDN1 in the resistance of colon cancer cells to chemotherapy. We show that high CLDN1 expression in liver metastases of CRC patients was associated with a poor response to chemotherapy and that membrane expression of CLDN1 was induced by chemotherapy in colon cancer cell models. Using a new antibody-drug conjugate (ADC) in which the CLDN1 antibody was coupled to MMAE we could inhibit the growth of CRC cell lines in vitro and in vivo. Interestingly, this effect was potentiated by a pre-treatment of the cells with oxaliplatin which is in accordance with the “one-two punch” approach that was previously described [20]. Together our results reinforce the role of CLDN1 in the acquired resistance to chemotherapy used in the treatment of CRC and validates the use of our anti-CLDN1 ADC as an alternative for the treatment of refractory CRC patients.

## Materials and methods

### Patients

The 89 liver metastasis specimens from 58 patients with CRC were prospectively collected at the Clinical and Biological Database BCB COLON (Institut du Cancer de Montpellier-Val d’Aurelle, France, Clinical trial Identifier #NCT03976960) following liver resection after preoperative chemotherapy regimens (chemotherapy alone or chemotherapy plus targeted therapies). Formalin-fixed paraffin-embedded tumor samples and data about the pathologic response were available for all included patients. Metastasis specimens without residual cancer cells were excluded. The histological response was evaluated with two scores: the histological TRG scoring system (based on the presence of residual tumor cells and fibrosis extent) [21] and the Blazer score (major response: -1-49% of residual cancer cells; minor response: ≥50% of residual cancer cells) [22]. For patients with more than one metastasis, the mean percentage of residual cancer cells was calculated to obtain the overall histological response. Moreover, as untreated control group, 26 liver metastasis specimens from 26 patients with CRC who underwent surgery at Montpellier Cancer Institute between 2002 and 2012 were selected (samples provided by the biological resource center; biobank number BB-0033-00059).

### Immunohistochemistry procedure

Three-µm-thin sections from the selected formalin-fixed paraffin-embedded tissue blocks were dried at 42 °C for 1 h before simultaneous deparaffinization, rehydration and antigen retrieval (at 95 °C for 15 min) in High pH buffer (Dako-Agilent, Glostrup, Denmark) using the PTLink module (Dako-Agilent). All subsequent steps were performed on a Dako Autostainer Link 48 platform (Dako-Agilent, Glostrup, Denmark). Endogenous peroxidase was quenched using Flex Peroxidase Block (Dako-Agilent) at room temperature for 5 min. Slides were then incubated with an anti-claudin-1 rabbit polyclonal antibody (Thermo Scientific, Ref 51-9000) at 1/100 at room temperature for 40 min. After 2 rinses in buffer, slides were incubated with a mouse anti-rabbit linker (Dako-Agilent) and then with a horseradish peroxidase-labeled polymer coupled to secondary anti-mouse and anti-rabbit antibodies for 30 min. The signal was revealed by incubation with 3,30-diaminobenzidine for 10 min, followed by counterstaining with Flex Hematoxylin (Dako-Agilent). After washing in tap water for 5 min and dehydration, slides were mounted with permanent mounting medium.

**Fig. 1:**
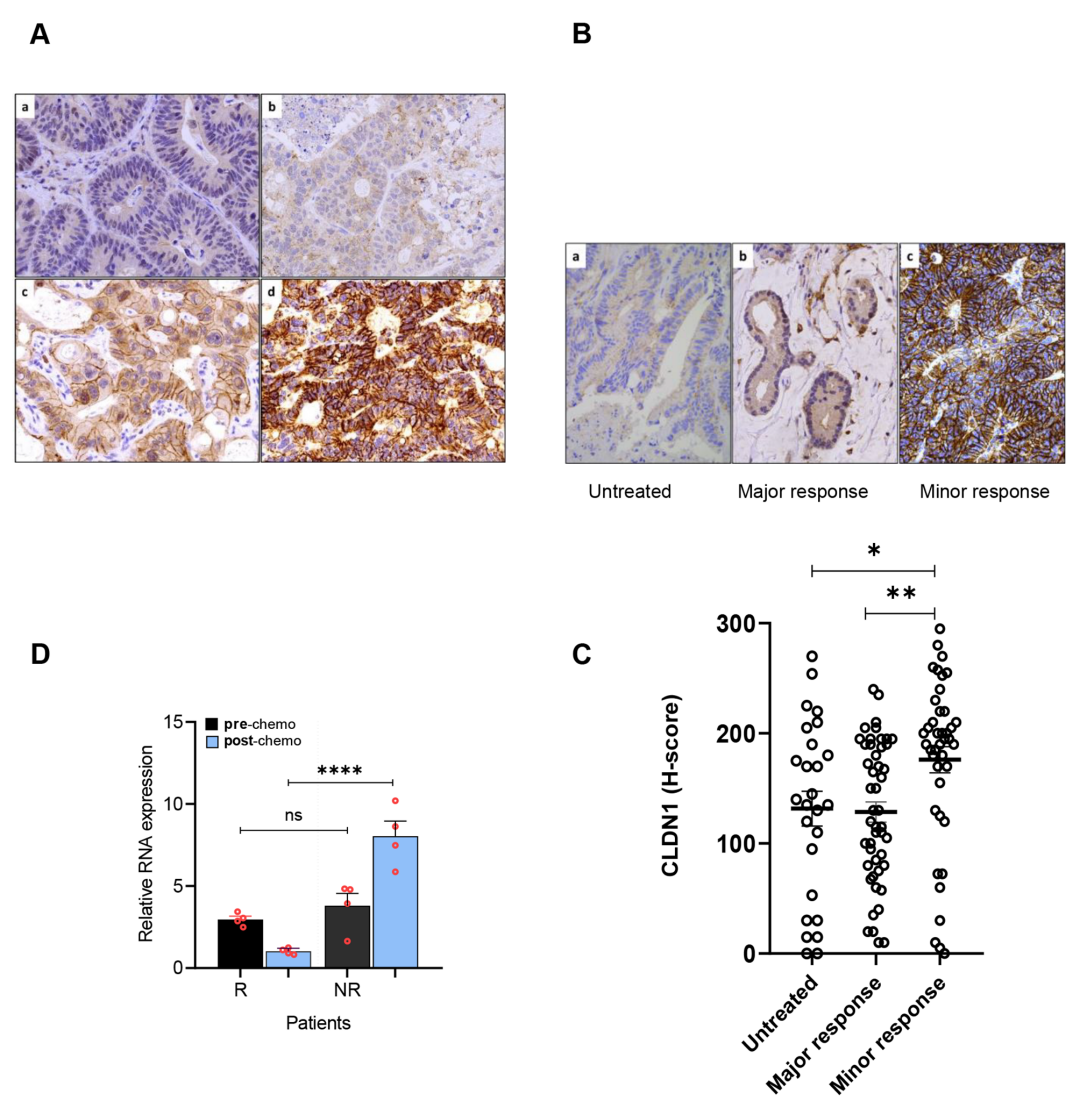
Immunohistochemical analysis of CLDN1 expression in post-chemotherapy colorectal cancer liver metastasis specimens. **(A)** Representative images of liver metastases from colorectal cancers after chemotherapy with various CLDN1 expression levels: no detectable signal (a), weak (b), moderate (c), and strong signal intensity (d); immunoperoxidase x400. **(B)** CLDN1 membrane expression in a non-treated metastasis (a), in residual tumor cells of liver metastasis specimens that displayed minor response (b) and major response (c) to chemotherapy; immunoperoxidase x400. **(C)** Comparison of CLDN1 expression in untreated (n = 26), major response (n = 48) and minor response (n = 41) metastasis specimens from patients with colorectal cancer. * p = 0.018 and **p = 0.002 (Kruskal-Wallis test). **(D)** CLDN1 RNA expression before/after chemotherapy in metastasis specimens from one responder (R) and one non-responder (NR) patients with colorectal cancer according to the RECIST response criteria: R (-87%), NR (0%). Quantitative RT-PCR was performed twice on each of the two different RNA samples from the same metastatic biopsy both prior to treatment and after treatment (n = 4). ****p < 0.0001 (one-way ANOVA test)

For each sample, CLDN1 membrane staining intensity was scored as negative (score 0), weak (score 1), moderate (score 2), or strong (3) (Fig. 1A) and the percentage of positive cells was reported for each intensity. A histochemical score (H-Score), ranging from 0 to 300, was calculated as the sum of the percentage of positive tumor cells multiplied by the staining intensity.

### Cell culture and treatment

The human CRC lines HCT116, SW480, SW620, (from the American Type Culture Collection, USA) and Difi (kindly provided by C. Montagut, Department of Medical Oncology, Hospital del Mar, Barcelona, Spain) were grown in RPMI-1640 with 10% fetal calf serum and 2mmol/L L-glutamine at 37 °C in a humidified atmosphere with 5% CO_2_. The oxaliplatin-resistant clones (HCT116_ROX, SW620_ROX) were previously described [23]. Cells were routinely tested for mycoplasma contamination using the MycoAlert™ detection kit (Lonza, Basel, Switzerland). Oxaliplatin (5 mg/ml) and 5-FU (50 mg/ml) were from the ICM pharmacy. SN38, p38 MAPK (LY2228820) and Wnt/β-catenin (XAV-939) inhibitors were purchased from Selleckchem (Euromedex, Souffelweyersheim, France). Propidium iodide and benzonase were purchased from SIGMA (Saint Quentin Fallavier, France).

### Protein silencing by shRNA

For CLDN1 and GSK3β silencing, specific shRNA vectors were purchased from Vectorbuilder (VectorBuilder GmbH, Germany) with the puromycin or neomycin selection marker. Lentiviruses were produced by co-transfecting 293T cells (for lentiviral packaging) with 1 µg of shRNA, 1 µg of the gag-pol packaging vector, and 1 µg of envelope vector, using jetPRIME™ (Polyplus), according to the manufacturer’s instructions. Lentiviral particles were harvested and then used to infect 1 × 10^6^ HCT116_ROX and SW620_ROX cells with shCLDN1 or SW620 cells with shGSK3β for 48 h. Following shRNA transduction, cells were selected with puromycin or neomycin. In all cases, shRNA against luciferase (-shLUC) was used as negative control.

### Flow cytometry

Cells were detached with trypsin (Gibco™ Enzyme TrypLE Express/), and 1 million cells were recovered, washed with PBS and incubated with 1% PBS-BSA (1 h at 4 °C). After centrifugation (1200 rpm for 5 min) and aspiration of the solution, cells were incubated with the following primary antibodies at 10 µg/mL: anti-CLDN1 6F6 mAb [19], 6F6-ADC, and anti-pGSK3β (D85E12, Cell Signaling). After 1 h incubation at 4 °C, cells were washed with PBS and were incubated with the relevant secondary antibodies (anti-human FITC F9512 Sigma-Aldrich, 1:200; or anti-rabbit Alexa Fluor® 488, Invitrogen, 1:500). Cells incubated only with the secondary antibody served as a negative control. Fluorescence was measured using a flow cytometer (Gallios Beckman Coulter).

### Western blotting

Total cell lysates were obtained as previously described [24]. Briefly, cells (10,000 cells/µL) were washed with 1X PBS and directly lysed in Laemmli buffer (4% SDS, 20% glycerol, 1% 2-β mercaptoethanol, 0.004% bromophenol blue, 0.125 M Tris HCL) in the presence of benzonase (25 units/ mL). After denaturation at 95 °C for 5 min, protein extracts were separated on SDS-PAGE polyacrylamide gels and then transferred to nitrocellulose membranes (0.45 μm pore size, Biorad). Then, membranes were blocked in PBS/0.1% Tween-20/5% milk at room temperature for 1 h, and incubated overnight with primary antibodies at 4 °C under gentle agitation. After three washes with PBS/0.1% Tween-20, membranes were incubated with the relevant anti-species secondary antibody coupled to horseradish peroxidase at room temperature for 1 h. Immunoreactions were revealed by chemiluminescence (ECL RevelBlot Intense Kits) and visualized using the G-Box imaging system (Syngene, Fisher Scientific, Illkirch, France), or visualized and quantified with the LI-COR Imager (LI-COR Biosciences - GmbH). Protein expression levels were normalized to the loading control or to the total protein loaded using Revert™700 Total Protein Stain. The following primary antibodies were used: anti-p38 MAPK (D13E1, #8690), anti-phosphorylated 38 MAPK (Thr180/Tyr182) (D3F9, #4511), anti-GSK-3β (3D10, #9832), anti-phosphorylated GSK3β^Ser9^ (D85E12, Rabbit mAb #5558), anti-GAPDH (14C10, #2118) from Cell Signaling Technology (CST), anti-CLDN1 (#51-9000) and anti-CD71 ( H68.4, #13-6800) from Invitrogen, anti-β-catenin (E-5; sc-7963, Santa-Cruz Biotechnology). HRP-linked anti-mouse IgG (#7076) or anti-rabbit IgG (#7074) (Cell Signaling Technology) were used as secondary antibodies.

### Subcellular fractionation

Protein extraction was performed using the Mem-PER Plus Membrane Protein Extraction Kit according to the manufacturer’s instructions (Thermo Fisher Scientific). Subcellular fractions (10 µg/each) were loaded on 12% SDS-PAGE gels. Western blotting was done as described above.

### Immunofluorescence

3.10^4^ cells were plated on cover slips in 24-well plates. Two days later, during the logarithmic phase of growth, cells were incubated with 5µM oxaliplatin during 72 h. Then, cells were washed twice with PBS/1% Tween-20 and once with PBS. Cells were fixed by incubation in 3.7% p-formaldehyde in PBS for 20 min, washed twice with PBS, and permeabilized with PBS/0.5% Triton X-100 at room temperature for 15 min. After two washes with PBS, cells were incubated in PBS/2% BSA for 1 h, and then with primary antibodies at 37 °C for 90 min. Cells were washed twice with PBS/0.1% Tween-20 and once with PBS and incubated with the secondary antibody at 37 °C for 45 min. Cells were washed with PBS/0.1% Tween-20 three times and with PBS three times, followed by mounting with Everbrithe® with DAPI and analysis using an epifluorescence Zeiss Imager 2. The primary antibodies were: anti-CLDN1 6F6 [19], anti-non-phosphorylated (active) β-catenin (Ser33/37/Thr41) (D13A1, CST#8814), anti-β-catenin (D10A8, CST#8480). Secondary antibodies were anti-human IgG (Fc specific)-FITC (F9512, Sigma Aldrich) and anti- rabbit IgG A488 (CST#4412).

### DuoLink proximity ligation assay

To visualize interactions between p38 MAPK and GSK3β, a proximity ligation assay (PLA) was performed using the Duolink® Kit (Sigma-Aldrich®) according to the manufacturer’s instructions. The first step was as described above for the immunofluorescence experiments with two primary antibodies against rabbit GSK3β (HPA028017, Sigma-Aldrich) and mouse p38 alpha (F-9; sc-271,120, Santa Cruz), followed by incubation with a pair of oligonucleotide-labeled secondary rabbit and mouse IgG (Duolink® In SituPLA® Probes). These two secondary antibodies generate a signal only when the two probes are in close proximity (40 nm). The PLA signals were assigned using Duolink® In Situ Detection Reagents Orange (554 nm laser line). Slides were counterstained with DAPI, mounted and visualized as described above.

### TopFlash luciferase reporter assays

1.5 × 10^4^ SW620 or Difi cells were plated in 24-well plates, then co-transfected with the firefly luciferase reporter plasmid TOP-FLASH or FOP-FLASH and the Renilla luciferase vector phRG-TK at 1 µg/ml (kindly provided by Dr P. Blache, IRCM, Montpellier, France) and incubated with oxaliplatin (5 µg/ml). 72 h post-transfection, cells were collected and the luciferase assay was performed using the Dual Luciferase Reporter Assay System (Promega) according to the manufacturer’s instructions. The relative firefly luciferase activity was normalized to the Renilla luciferase activity as a control for transfection efficiency.

### RT-qPCR

Total RNA was extracted from cultured cells using the Quick-RNA Miniprep Kit (Zymo Research) following the manufacturer’s protocol. cDNA was synthesized using SuperScript III Reverse Transcriptase (Thermo Fischer Scientific). Quantitative PCR was performed using ONEGreen® Fast qPCR Premix (Ozyme) and the LightCycler® 480 Real-Time PCR System according to the manufacturer’s instructions (Roche). The relative mRNA expression levels were calculated using the 2-△△Ct method. All primers were synthesized by Eurofins Genomics and are listed in Supplementary Table 2.

### Cytotoxic assay (2D culture)

Cytotoxicity was tested using the sulforhodamine B (SRB) assay, as described by [25]. Briefly, 500–1000 cells/well were seeded in 96-well plates. Twenty-four hours later, exponentially growing cells were incubated with serial dilutions of oxaliplatin for 96 h. Cells were then fixed in trichloroacetic acid solution (final concentration of 10%), and stained with 0.4% SRB solution in 1% acetic acid (Sigma Chemical Co., USA). Fixed SRB was dissolved in 10mM Tris-HCl solution and absorbance at 560 nm was read using a Thermo Scientific Multiskan EX plate reader (USA). The IC_50_ was determined graphically from the cytotoxicity curves.

### Spheroid formation and cytotoxic assays

For spheroid formation, 500 SW620 cells were seeded in round-bottom plates (Corning® Costar® Ultra-Low Attachment Multiple Well Plate size 96 CLS700). After 3 days, spheroids were formed and were used for experiments. To evaluate ADC effects, spheroids were incubated with 6F6-ADC or control-ADC (10 µg/ml) for 7 days. Spheroid growth was monitored daily using the Celigo™ imaging cytometer by measuring the spheroid area with the “Tumorospheres” application. After 7 days, some spheroids were incubated with 1 mg/mL propidium iodide (PI) that emits red fluorescence once inside dead cells. After 30 min of incubation, PI staining intensity in dead cells within the spheroids was imaged using the Celigo™ imaging cytometer.

To evaluate the effect of the oxaliplatin + ADC sequential combination, spheroids were incubated with different concentrations of oxaliplatin (0-0.4µM), SN38 (0-2.5 nM), or 5-FU (0–4 µM) for 72 h. Then, spheroids were incubated with different 6F6-ADC concentrations (0–3 µg/mL) for 7 days. Cell viability was evaluated with the CellTiter-Glo® Luminescent Cell Viability Assay (Promega) according to the manufacturer’s recommendations. Luminescence was recorded using a luminometer (PHERAstar® FSX).

### Synergy matrix

The percentage of living cells after incubation with each drug alone or in combination is calculated. This percentage is normalized to untreated cells. Then, using a script in the “R” software based on the effect of each molecule alone (Bliss and Lehàr equation) [26], a synergy matrix is generated. The number associated with each combination, when positive, indicates the part of the observed effect due to the synergy; when negative, it indicates an antagonism between molecules. On the basis of the associated number, each combination is defined by a color that indicates the type of effect observed. Synergy is associated with the color red, additivity with black, and antagonism with green.

### RNA-seq

RNA from the different cell lines (SW20-ROX_shCLDN1 and -shLUC), incubated or not with oxaliplatin, were sent to Paris Brain Institute Data Analysis Core company for RNA-sequencing, using three replicates per group. The raw data quality was evaluated with FastQC. Poor quality sequences and adapters were trimmed or removed with the fastp tool, using default parameters, to retain only good quality paired reads. The Illumina DRAGEN bio-IT Platform (v3.8.4) was used for mapping to the hg38 reference genome and quantification with the Gencode vM25 annotation gtf file. Library orientation, library composition and coverage of transcripts were checked with the Picard tools. The following analyses were done with R. Data were normalized with the DESeq2 (v1.26.0) or/and edgeR (v3.28.0) bioconductor packages, prior to differential analysis with the glm framework likelihood ratio test from the edgeR package, and/or the DESeq2 workflow. Adjusted p-values for multiple hypotheses were calculated with the Benjamini-Hochberg procedure to control FDR. Finally, the enrichment analysis was performed with the clusterProfiler R package (v3.14.3) with over-representation analysis and/or Gene Set Enrichment Analysis and the gene ontology database.

### Apoptosis analysis with annexin V/7AAD

2 × 105 oxaliplatin-resistant SW620_ROX, HCT116_ROX cells in which CLDN1 was silenced (-shCLDN1) or not (-shLUC) were plated, and after 24 h, they were incubated with oxaliplatin at 5µM for 24 h. Cells were stained with fluorescein isothiocyanate (FITC)-labeled annexin V and 7-Amino-Actinomycin D (7-AAD) (Annexin V/7-AAD - Beckman Coulter). For apoptosis determination, annexin V- and 7-ADD-positive cells were quantified using a NucleoCounter® NC-3000™ (ChemoMetec Inc) according to the manufacturer’s instructions.

### ADC design

The linker diSPh-PEG12-VC-MMAE contain the VC-PABC-MMAE sequence, with VC (valine-citrulline) as the cathepsin B-sensitive trigger, and PABC (para-aminobenzyl carbamate) as a self-immolative spacer to free the cytotoxic MMAE [27]. As MMAE is quite hydrophobic, it can diffuse through biological membranes to exert a bystander killing effect. A site-specific method was used to conjugate linkers to the antibodies 6F6 or Control using a (diphenylthiomaleimido)caproic acid (diSPhMC) chemical entity for disulfide stapling on cysteine residues. This method allows the re-bridging of previously reduced interchain disulfide bonds, leading to stable and homogeneous ADCs [28–30]. (see supplemetal information for the detailed ADC generation procedure). To generate ADC control, we used chimeric anti-CD20 mAb (isotype IgG1k) for in vitro and Human IgG1, kappa Isotype Control (Sino Biological) for in vivo experiments.

### In vivo tumor cell xenograft assays

1 × 10^6^ SW620 cells were suspended in culture medium and Matrigel (v/v) and injected subcutaneously into the right flank of 6-week-old female athymic nude mice (Charles River Laboratories, France). When the tumor volume reached approximately 100 mm^3^, mice were randomized in different groups. For kinetic experiments, mice were treated at day 0 and day 9 with oxaliplatin (2 mg/kg). At days 3, 7, 12 and 16, tumors were removed, dissociated and analyzed by flow cytometry. For ADC experiments, mice received by iv injection 0.9% NaCl or ADCs (5 mg/kg per injection) twice per week for 4 weeks. For the sequential combination experiments, mice received oxaliplatin at 3 mg/kg once per week followed by an iv injection of ADC-6F6 at 5 mg/kg after 3 and 6 days. Tumors were detected by palpation and were measured with a caliper twice per week. Tumor volumes calculated with the formula: D1 x D2 x D3/2.

### Statistical analyses

They were performed using PRISM version 8.0 and GraphPad (San Diego, CA, USA) for in vitro assays. Data are the mean ± SEM and the two-tailed Student’s *t* or Mann-Whitney tests and 1-way ANOVA or Kruskal-Wallis tests (for more than two groups) were used to calculate p-values. To test the relationships between clinical variables and CLDN1 expression, the Chi-square test or Fisher’s exact test were used. For in vivo experiments, statistical analyses were performed with the STATA 16 software (Stata Corporation, College Station, TX, USA). A linear mixed regression model was used to determine the relationship between tumor growth and number of days after injection. Survival rates were estimated from the date of the injection until the date when the tumor reached the volume of 1500 mm^3^ using the Kaplan–Meier method. Survival curves were compared using the log-rank test. Differences were considered statistically significant at * *p* < 0.05** *p* < 0.01, *** *p* < 0.001 and **** *p* < 0.0001.

## Results

### High CLDN1 expression is correlated with poor histologic response to chemotherapy in patients with CRC

To evaluate the potential CLDN1 implication in chemoresistance development, we first asked whether its expression was correlated with the pathologic response to preoperative chemotherapy. For this, we analyzed by immunohistochemistry the membrane expression of CLDN1 in 89 post-chemotherapy CRC liver metastasis specimens from 58 patients. As CLDN1 expression varied among samples (Fig. 1A), we used a CLDN1 H-score to measure CLDN1 expression in each specimen and we calculated the mean CLDN1 H-score for patients with more than one metastasis. To assess the correlation between CLDN1 expression and clinical characteristics, we dichotomized the 58 patients in two groups based on the median CLDN1 H-score = 169 (n = 29 patients per group). We found that CLDN1 expression was significantly correlated with the two conventional criteria of histological assessment [21, 22]: Blazer score (p = 0.029) and tumor regression grading (TRG) (p = 0.017) (Table 1). To precisely analyze this correlation, we classified the 89 metastasis samples in two groups: minor response and major response, in function of the percentage of residual cancer cells after chemotherapy (Blazer score = major response: -1-49% of residual cancer cells, and minor response: ≥50%) (Fig. 1B). CLDN1 expression (H-score) was significantly higher in the minor response group (n = 41 specimens) than in the major response group (n = 48 specimens) (p = 0.002) (Fig. 1C). Furthermore, CLDN1 expression was significantly higher in the minor response group than in 26 untreated liver metastases (p = 0.018). On the other side, the H-score was comparable between untreated metastases and major response group (Fig. 1C). In agreement, using pre- and post-chemotherapy metastases samples from two patients (such samples originate from a previous study [31] and are rather difficult to obtain in clinical practice), we could show that CLDN1 mRNA level was significantly higher after chemotherapy in metastasis from non-responder patient than responder patient (according to the RECIST criteria) whereas it was similar on samples collected before chemotherapy (Fig. 1D). Together, these findings indicate a strong correlation between CLDN1 expression and histological response to systemic treatment, suggesting that acquisition of chemoresistance may, at least in part, be explained by chemotherapy-induced CLDN1 overexpression.

**Table 1:** Clinical and pathological characteristics of patients

CLDN1 expression	< median		> median		total		p-value***
	(n = 29)		(n = 29)		(n = 58)		
	N	(%)	N	(%)	N	(%)	
**Gender**							
Male	22	(75.9)	13	(44.8)	35	(60.3)	*0.03*
Female	7	(24.1)	16	(55.2)	23	(39.7)	
**Age (years)**, median [63]							
≤ 63	11	(38.0)	18	(62.1)	29	(50.0)	*0.07*
> 63	18	(62.0)	11	(37.9)	29	(50.0)	
**Primary tumor localization**							
Right colon	8	(27.6)	12	(41.4)	20	(34.5)	*0.31*
Left colon	20	(68.9)	17	(58.6)	37	(63.8)	
Missing data	1	(3.4)	0	(0.0)	1	(1.7)	
**Differentiation**							
Well	10	(34.5)	8	(27.6)	18	(31.0)	*0.38*
Moderate	15	(51.7)	20	(69.0)	35	(60.3)	
Missing data	4	(13.8)	1	(3.4)	5	(8.7)	
**Synchronous**	17	(58.6)	24	(82.7)	41	(70.7)	*0.08*
**Metachronous**	12	(41.4)	5	(17.3)	17	(29.3)	
**Chemotherapy regimen**							
FOLFIRI	5	(17.3)	8	(27.6)	13	(22.4)	*0.58*
FOLFOX	13	(44.8)	10	(34.5)	23	(39.7)	
FOLFIRINOX	11	(37.9)	11	(37.9)	22	(37.9)	
**Targeted therapy**							
YES	6	(20.7)	11	(37.9)	17	(29.3)	*0.15*
NO	23	(79.3)	18	(62.1)	41	(70.7)	
**Number of cures**,							
≤ 6	17	(58.6)	19	(65.5)	28	(48.3)	*0.56*
> 6	11	(37.9)	9	(31.0)	28	(48.3)	
Missing data	1	(3.5)	1	(3.5)	2	(3.4)	
**KRAS status**							
WT	7	(24.1)	10	(34.5)	17	(29.3)	*0.74*
Mutated	12	(41.4)	14	(48.3)	26	(44.8)	
Missing data	10	(34.5)	5	(17.2)	15	(25.9)	
**Histological response**							
Blazer score*							
1	18	(62.0)	9	(31.0)	27	(46.6)	***0.029***
2	10	(34.5)	17	(58.6)	27	(46.6)	
Missing data	1	(3.5)	3	(10.4	4	(6.8)	
TRG**							
2	6	(20.7)	1	(3.5)	7	(12.0)	***0.017***
3	12	(41.3)	5	(17.2)	17	(29.3)	
4	10	(34.5)	19	(65.5)	29	(50.0)	
5	0	(0.0)	1	(3.5)	1	(1.7)	
Missing data	1	(3.5)	3	(10.3)	4	(7.0)	

* Blazer score (1 = major response: 1–49% of residual cancer cells; and 2 = minor response: ≥50% of residual cancer cells).**TRG = scoring system based on the presence of residual tumor cells and fibrosis extent.*** Chi-square or Fisher’s exact test

### Chemotherapy induces overexpression of membrane CLDN1 in CRC cells

**Fig. 2:**
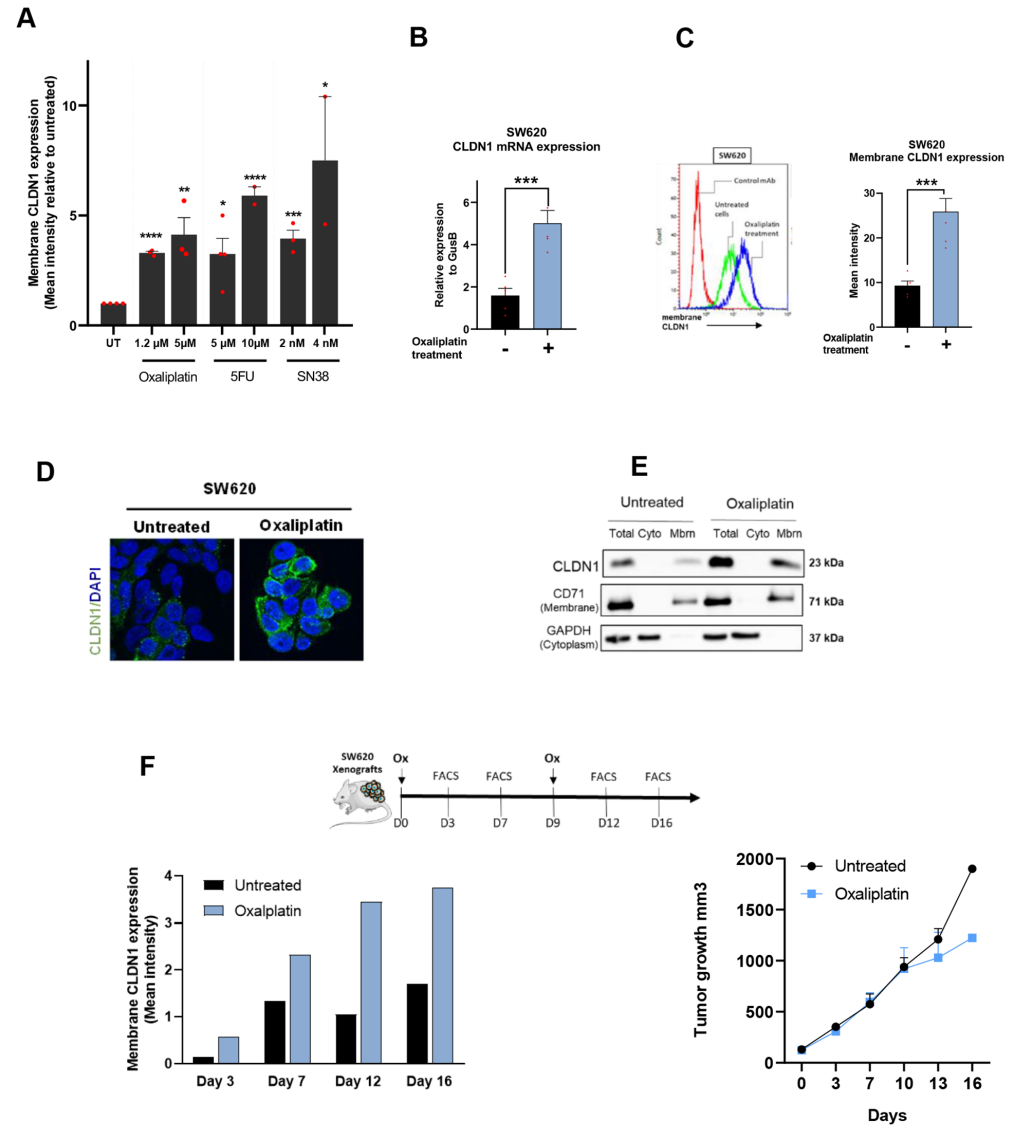
CLDN1 is overexpressed at the membrane of colorectal cancer cells after exposure to chemotherapy drugs. **(A)** Flow cytometry analysis of CLDN1 expression at the surface of SW620 cells incubated with conventional chemotherapy agents (5-FU, SN38, oxaliplatin) at two concentrations. * p ≤ 0.05, ** p ≤ 0.01, *** p ≤ 0.001, **** p ≤ 0.0001 (Student’s t-test). **(B)** Relative CLDN1 gene expression in SW620 cells before (-) and after (+) incubation with oxaliplatin (1.2 µM for 72 h); *** p ≤ 0.001 (Student’s t-test). **(C)** Membrane CLDN1 expression analyzed by flow cytometry in SW620 cells before (-) and after (+) incubation with oxaliplatin (1.2 µM for 72 h); *** p ≤ 0.001 (Student’s t-test). **(D)** Immunofluorescence analysis of CLDN1 membrane expression in SW620 cells before (-) and after (+) incubation with oxaliplatin (1.2 µM for 72 h) **(E)** Subcellular localization of CLDN1 by western blotting in SW620 cells before (-) and after (+) incubation with oxaliplatin (1.2 µM for 72 h). CD71 and β-actin were used as markers for the membrane and cytoplasm fractions, respectively. Cyto, cytoplasm; Mbm, membrane. **(F)** In vivo kinetics of CLDN1 expression at the tumor cell membrane. Top: experimental setup: mice xenografted with SW620 cells were treated at day 0 and day 9 or not with oxaliplatin (Ox; 2 mg/kg). At the indicated time points, two tumors per condition were removed, dissociated and analyzed by flow cytometry. Left: Histograms showing CLDN1 membrane expression (g-mean) in untreated (black) and oxaliplatin-treated (blue) tumors. Right: tumor growth curves in untreated and oxaliplatin-treated mice xenografted with SW620 cells

To test this hypothesis, we assessed the effect of single-agents used in the FDA-approved FOLFOX or FOLFIRI combinations for CRC (5-FU, irinotecan, and oxaliplatin) on CLDN1 expression in the SW620 CRC cell line. Flow cytometry results showed that the three drugs strongly increased CLDN1 expression at the cell membrane in a dose-dependent manner (Fig. 2A). We then focused on oxaliplatin to unravel the mechanism of CLDN1 induction. Incubation with oxaliplatin showed a marked and significant 2.5-fold increase of CLDN1 mRNA (p ≤ 0.001) and protein expression (p ≤ 0.001) in the CLDN1-positive SW620 CRC cell line (Fig. 2B-C). We obtained similar results in two other CRC cell lines, HCT116 (CLDN1-weak) and Difi (CLDN1-positive), regardless of their basal CLDN1 expression level (Supplementary Fig. 1A-B). We confirmed the oxaliplatin-mediated CLDN1 overexpression at the cell membrane in SW620 cells by immunofluorescence analysis and subcellular fractionation (Fig. 2D-E). In addition, these results were confirmed in vivo in mice xenografted with SW620 cells. From day 3 after oxaliplatin treatment (2 mg/kg), CLDN1 membrane expression increased progressively and more strongly in treated than untreated mice, independently of the tumor growth (Fig. 2F). Altogether, these findings indicate that CLDN1 expression (mRNA and protein) in CRC cells is upregulated by chemotherapy in vitro and in vivo, and suggest that CLDN1 may have an active role in the resistance to chemotherapy.

### Oxaliplatin increases membrane CLDN1 expression via the p38/GSK3β/Wnt-β-catenin pathway

**Fig. 3:**
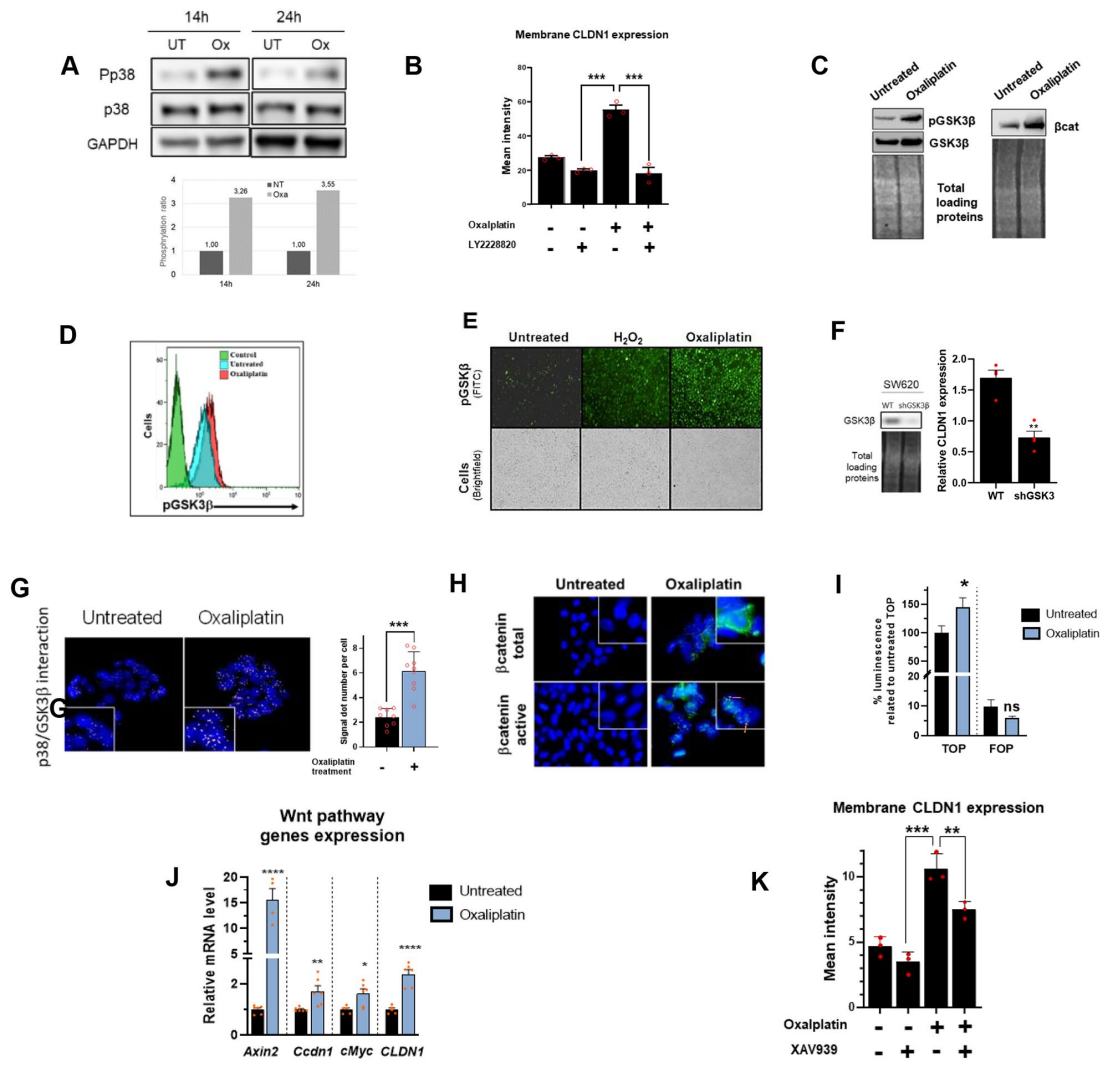
Oxaliplatin-mediated membrane CLDN1 expression is dependent on MAPKp38,/GSK3β/ Wnt-βcat signaling cascade **(A)** Analysis of MAPK p38 phosphorylation (Pp38) by western botting in SW620 cells after incubation or not (UT) with oxaliplatin for 14 and 24 h (1.2 µM) Top: representative western blotting image; bottom: quantification of MAPK p38 phosphorylation level in the different conditions. **(B)** CLDN1 expression at the membrane (g-mean quantification) of SW620 cells incubated (+) or not (-) with oxaliplatin and/or LY2228820 (p38 inhibitor) (1 µM) for 72 h p ≤ 0.001 (Student’s t-test). **(C)** Western blotting showing the expression of GSK3β, GSK3βSer9 and β-catenin in SW620 cells incubated or not with oxaliplatin (5µM for 24 h). (D-E) GSK3βSer9 expression evaluated **(D)** by flow cytometry and **(E)** by immunofluorescence using the Celigo™ imaging cytometer. Incubation with H2O2 was used as positive control because it increases GSK3β phosphorylation at Ser9 (20). **(F)** GSK3β silencing. Left, western blot analysis of GSK3β expression in SW620 cells in which GSK3β was silenced (shGSK3β) or not (WT). Right, effect of GSK3β silencing on CLDN1 membrane expression after incubation with oxaliplatin (1.2 µM for 72 h) evaluated by FACS relative to non-silenced cells ** p = 0.001 (Student’s t-test). **(G)** Proximity ligation assay (PLA) with oligonucleotide-conjugated antibodies against p38 and GSK3β in SW620 cells incubated or not with oxaliplatin (5µM for 24 h). Left: Fluorescence images, nuclei were counterstained with DAPI (blue). Right: PLA dot counts per cell in the corresponding fluorescence images p ≤ 0.001 (Student’s t-test) **(H)** Expression and localisation of total and inactive (phosphorylated) βcatenin by immunofluorescence staining in SW620 cells after incubation or not with oxaliplatin (5µM for 72 h). In treated cells, total β-catenin expression increases and inactive β-catenin translocates to the nucleus (arrow) **(I)** The TOP/FOP Flash luciferase assay shows the transcriptional activation of the Wnt/β-catenin signaling pathway in SW620 cells after oxaliplatin incubation (5µM for 72 h) compared with untreated cells. * p = 0.01 (paired t-test) **(J)** Oxaliplatin (5µM for 72 h) effect on the mRNA expression of Wnt /β-catenin target genes in SW620 cells compared with untreated cells (Student’s t-test). **(K)** Oxaliplatin-induced (1.2µM for 72 h) CLDN1 membrane expression increase is reduced when SW620 cells are co-incubated with 7.5 µM XAV939 (small molecule inhibitor of tankyrase) ****p < 0.0001 (one-way ANOVA test)

To determine the molecular pathway(s) involved in oxaliplatin-mediated CLDN1 overexpression, we first focused on the p38 mitogen-activated protein kinase (MAPK) signaling for two reasons: (i) p38 MAPKs have a role in transducing stress signals from the environment [32] and (ii) they are activated by chemotherapy in CRC cell lines [33]. After incubation of SW620 cells with oxaliplatin, p38 activating phosphorylation was increased by more than 3-fold compared to untreated cells (Fig. 3A). Moreover, CLDN1 expression increase was associated with p38 activation since its pharmacological inhibition using LY228820 inhibitor prevented its induction (Fig. 3B). Then, to identify downstream effectors, we performed a phospho-protein array experiment. We found that in oxaliplatin-treated SW620 cells, GSK3β phosphorylation at Ser9 and β-catenin expression were increased compared with untreated cells (Supplementary Fig. 2A). We confirmed this result by western blotting (both proteins) (Fig. 3C), by FACS and immunofluorescence analysis of GSK3β phosphorylation using H_2_0_2_ as a positive control [34] (Fig. 3D-E). To investigate GSK3β implication in oxaliplatin-mediated CLDN1 overexpression, we silenced GSK3β by shRNA in SW620 cells. When GSK3β expression was downregulated, oxaliplatin could not induce the expression of CLDN1 (Fig. 3F). As p38 can associate with and phosphorylate GSK3β [35], we used a proximity ligation assay to assess this interaction in our model. We found that p38 and GSK3β colocalized in CLDN1-positive SW620 and Difi cells. The colocalization signal was significantly amplified upon incubation with oxaliplatin (Fig. 3G and Supplementary Fig. 2B), suggesting an interaction between p38 and GSK3β. Then, we investigated β-catenin activation (dephosphorylated form) and localization in SW620 and Difi cells after incubation with oxaliplatin. In both cell lines, total β-catenin was increased as well as the translocation of active β-catenin in the nucleus (Fig. 3H and Supplementary Fig. 2C). We therefore performed a TOP/FOP luciferase reporter assay to monitor β-catenin transcriptional activity in the nucleus of SW620 and Difi cells. The nuclear transcriptional activity of β-catenin was significantly increased by oxaliplatin treatment in both cell lines (Fig. 3I and Supplementary Fig. 2D).

The deregulation of GSK3β and β-catenin, two important players of the Wnt/β-catenin pathway, led us to assess the activation of the Wnt/β-catenin signaling pathway by monitoring the expression of its target genes. After oxaliplatin incubation, *AXIN2*, *Cyclin D1* (*CCDN1*) and *c-Myc* (*MYCBP*), three well-known Wnt/β-catenin target genes [36], were upregulated concomitantly with *CLDN1*, which is regulated by β-catenin [37] (Fig. J and Supplementary Fig. 2E). Lastly, inhibition of the Wnt/β-catenin pathway with XAV939 (a small molecule inhibitor of tankyrase) prevented the increase of CLDN1 expression at the cell membrane upon oxaliplatin incubation (Fig. 3K).

These findings suggest that following incubation with oxaliplatin, p38 is activated by phosphorylation and phosphorylates GSK3β at Ser9 (inactivating phosphorylation). GSK3β^ser9^ cannot phosphorylate β-catenin, leading to its stabilization, translocation to the nucleus, activation of the Wnt/β-catenin pathway and ultimately increased induction of CLDN1 membrane expression.

### CLDN1 is a key player in oxaliplatin resistance in CRC cell lines

**Fig. 4:**
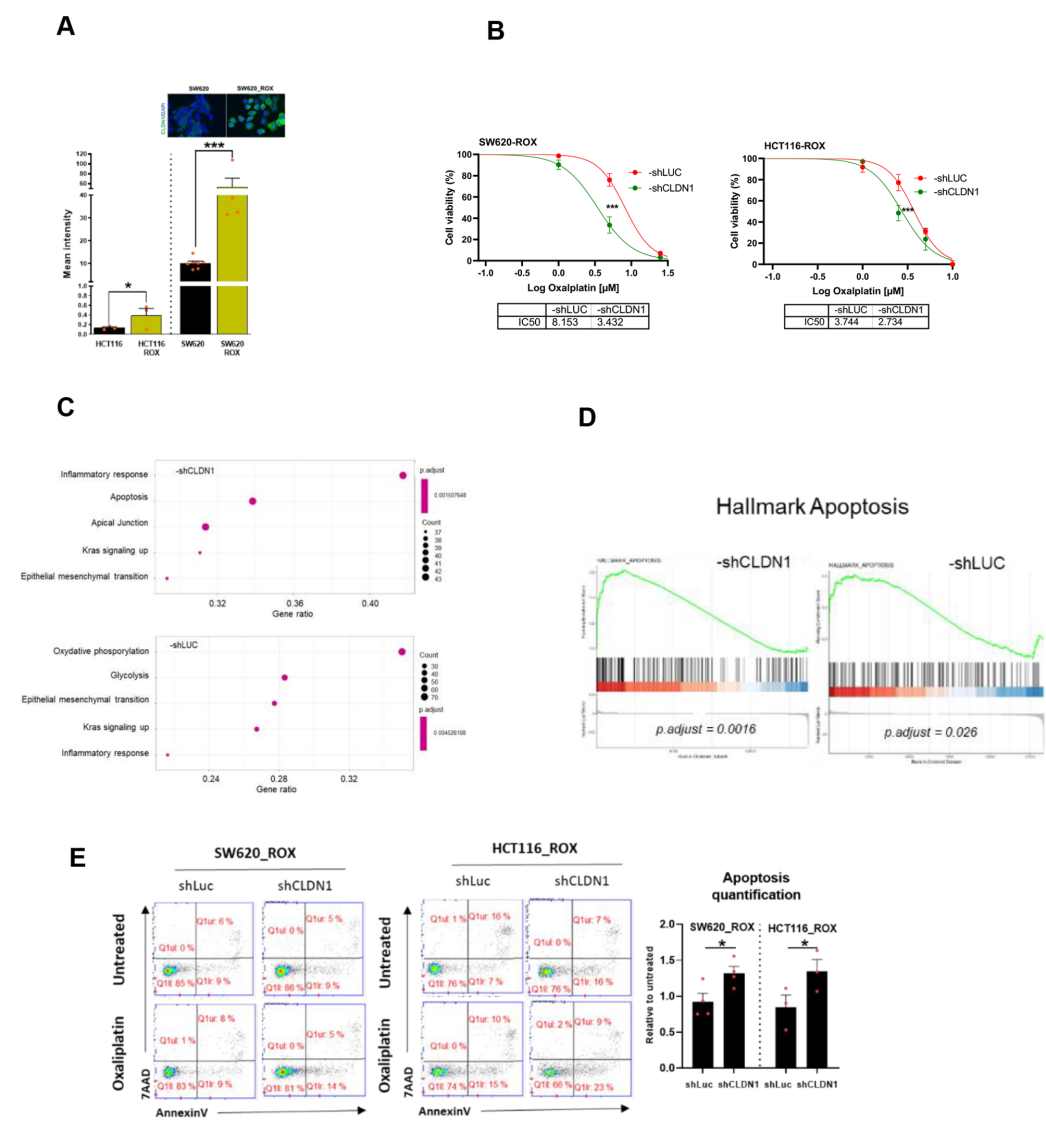
CLDN1 is a key mediator to oxaliplatin resistance in CRC cell lines **(A)** CLDN1 membrane expression in oxaliplatin-resistant cell lines and their parental cell lines. Top: Immunofluorescence images of CLDN1 membrane expression in SW620-ROX cells (oxaliplatin resistant) and in SW620 cells (parental line). **(B)** Effect of CLDN1 silencing (shCLDN1) on oxaliplatin IC50 in the two oxaliplatin-resistant cell lines incubated with oxaliplatin compared with the shLUC controls. **(C)** Five GSEA hallmarks deregulated after oxaliplatin treatment with significantly enriched genes in SW620_ROX–shCLDN1 and -shLUC cells. Dot plots indicate the gene ratios (number of core genes over the total number of genes in the set). Dots are colored in function of the adjusted p-value and their size in the gene set. **(D)** Enrichment plots for the apoptosis hallmark. The location of the gene set members is indicated by vertical black lines and showed a significant positive enrichment (left) in SW620_ROX–shCLDN1 cell samples compared with SW620_ROX-shLUC samples **(E)** FACS profiles of annexin V-APC/7-AAD staining in the two oxaliplatin-resistant cell lines in which CLDN1 was silenced (-shCLDN1) or not (-shLUC) and incubated or not with oxaliplatin (5µM for 24 h). Apoptosis was quantified in at least three experiments (Student’s t-test)

To determine the CLDN1 role in chemoresistance of CRC cells, we evaluated its expression in our established oxaliplatin-resistant cell lines [23]. CLDN1 expression at the membrane was significantly higher in oxaliplatin-resistant cell lines than in the parental cell lines, regardless of CLDN1 basal level (Fig. 4A). We further confirmed this finding in the oxaliplatin-resistant SW620_ROX cell line that strongly expresses CLDN1. To determine CLDN1 implication in the resistance to oxaliplatin, we evaluated the sensitivity of SW620_ROX and HCT116_ROX resistant cells in which the expression of *CLDN1* was stably downregulated using shRNA (shLUC was used as negative control) (Supplementary Fig. 3). We found that CLDN1 silencing increased the cell sensitivity to oxaliplatin in a dose-dependent manner, as indicated by the reduction of the IC_50_ values by 2.4-fold and 1.4-fold in SW620-ROX_shCLDN1 (p < 0.0001) and HCT116-ROX_shCLDN1 cells (p = 0.0004), respectively, as compared with shLUC cells (Fig. 4B). This suggests that CLDN1 could be implicated in oxaliplatin resistance.

To identify the molecular pathways underlying oxaliplatin resistance mediated by CLDN1, we performed RNA-seq analyses in SW620-ROX_shCLDN1 and SW620-ROX_shLUC cells incubated or not with oxaliplatin. Principal component analysis (PCA) data showed that SW620-ROX_shCLDN1 and SW620-ROX_shLUC were clustered differently based on their global transcriptome profiles (Supplementary Fig. 4A). Differential analysis of treated and untreated samples highlighted 516 deregulated genes (376 down- and 140 up-regulated) in SW620-ROX_shCLDN1 cells, and 665 deregulated genes (519 down-, 146 up-regulated) in SW620-ROX_shLUC cells (FDR < 0.05) (Supplementary Fig. 4B). Gene set enrichment analysis (GSEA) using the hallmark gene sets identified 23 and 15 modules enriched in SW620-ROX_shCLDN1 and SW620-ROX_shLUC cells, respectively (adjusted p < 0.05) (Supplementary Table 1). The most significant hallmark modules were inflammatory response, apoptosis, apical junction, KRAS signaling up, and epithelial mesenchymal transition for SW620-ROX_shCLDN1 cells (adjusted p < 0.002), and oxidative phosphorylation, glycolysis, epithelial mesenchymal transition, KRAS signaling up, and inflammatory response for SW620-ROX_shLUC cells (adjusted p < 0.005) (Fig. 4C). Upon incubation with oxaliplatin, genes related to the inflammatory response, epithelial-mesenchymal transition, and KRAS pathway activation were enriched in both SW620-ROX_shCLDN1 and SW620-ROX_shLUC cell lines, indicating their oxaliplatin-dependent activation. Metabolic genes involved in glycolysis and oxidative phosphorylation were strongly downregulated in SW620-ROX_shLUC cells. Conversely, genes encoding adhesion and tight junction proteins were only enriched and upregulated in SW620-ROX_shCLDN1 cells (Supplementary Fig. 4C).

**Fig. 5:**
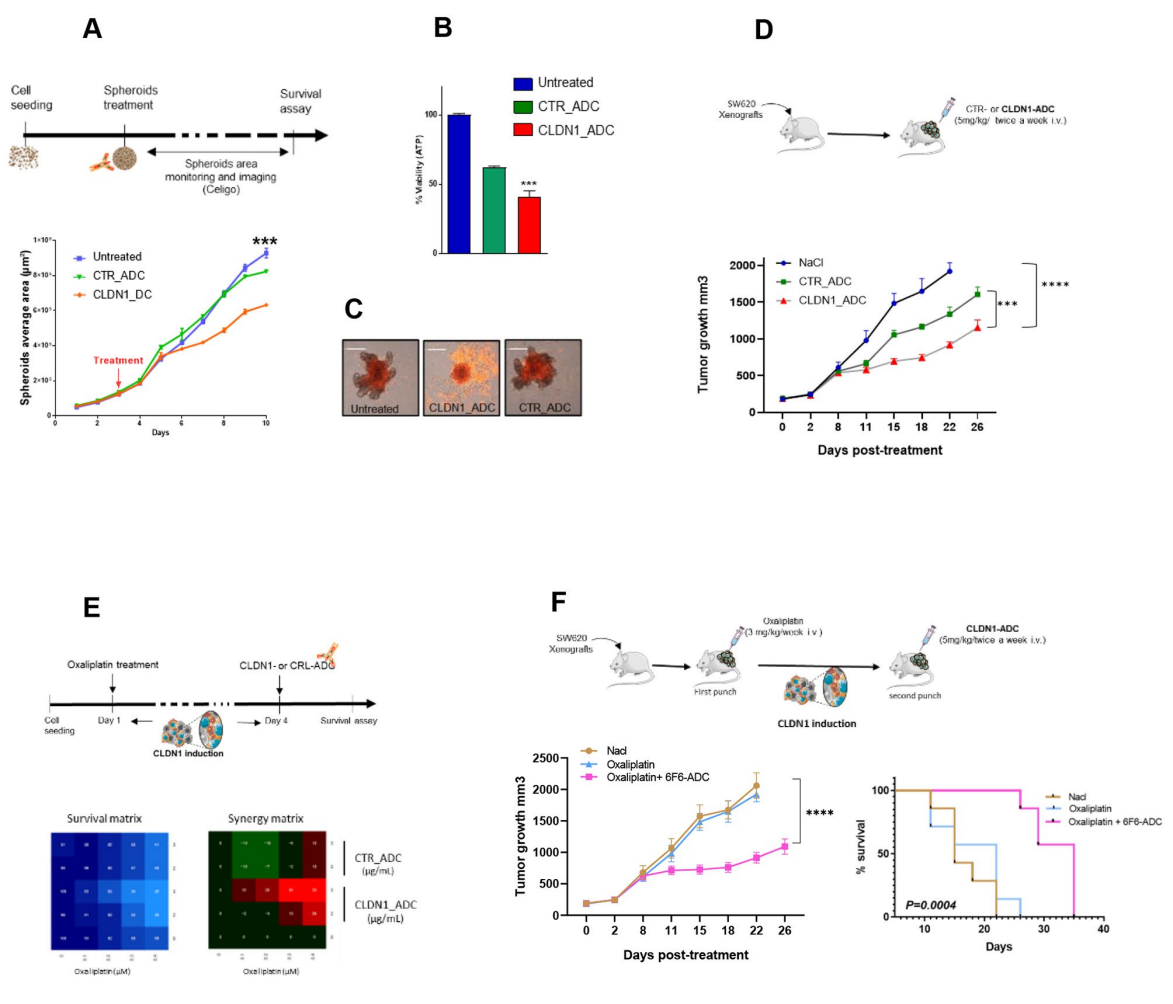
Therapeutic effect of an anti-CLDN1 ADC in colorectal cancer cells and “one-two punch” therapeutic approach. **(A)** Growth curve of SW620 cell spheroids incubated with 10 µg/mL of 6F6-ADC, Control-ADC (anti-CD20 mAb), or not for 7 days monitored with the Celigo™ imaging cytometer. **(B)** Cell survival in spheroids at the experiment end was determined with a cytotoxicity assay. The luminescence intensity (i.e. viable cells) was measured and compared in the three conditions described in (A). **(C)** Spheroids were incubated with 1 µg/m of propidium iodide (PI) that emits a red fluorescence red when incorporated in dead cells. Images were acquired using the Celigo™ imaging cytometer. **(D)** Effect of 6F6–ADC and control-ADC (Human IgG1, kappa Isotype Control) on the growth of SW620 cell xenografts in athymic nude mice. Mice were treated or not (blue) with 5 mg/kg of Control-ADC (green) or 6F6-ADC (red) per week starting when tumors reached 100 mm3 (n = 7 mice per group). **(E)** In vitro analysis of the effects of the oxaliplatin + 6F6-ADC combination in SW620 cells. At 24 h post-seeding cells were incubated with increasing doses of oxaliplatin and 72 h later with increasing doses of 6F6-ADC or control-ADC. One week later, the cell viability assay was performed. The blue matrix represents cell viability. In the synergy matrix, red, black, and green represent synergy, additivity, and antagonism, respectively. **(F)** Tumor growth curves in mice xenografted with SW620 cells untreated, treated with oxaliplatin alone or with the sequential combination of oxaliplatin and 6F6-ADC (7 mice per group). Adapted Kaplan-Meier curves using the time taken to reach a tumor volume of 1500 mm3 in untreated, oxaliplatin-treated and sequential combination-treated mice (log-rank test)

Moreover, SW620-ROX_shCLDN1 cells were more enriched in upregulated programmed cell death and caspase pathway genes (Fig. 5D), particularly the two pro-apoptotic genes *TXNIP* and *GADD45B* (Supplementary Fig. 4D). In agreement, incubation with oxaliplatin resulted in increased apoptosis in both SW620-ROX_shCLDN1 and HCT116-ROX_shCLDN1 cells compared with the shLUC controls (Fig. 4E). This shows that CLDN1 downregulation promotes apoptosis in oxaliplatin-resistant CRC cell lines and suggests an anti-apoptotic role for CLDN1.

### Therapeutic effect of an anti-CLDN1 ADC in CRC cells

We previously produced a mAb against the extracellular part of CLDN1 (6F6), and showed its cytostatic therapeutic efficacity in different CRC models [19]. To increase its therapeutic efficacity, we generated an ADC (6F6_ADC) by coupling the 6F6 mAb with the cytotoxic antimitotic agent monomethyl auristatin E (MMAE) [38, 39]. The comparison between the naked 6F6 antibody and the 6F6-ADC was performed on SW620 and DIFI cell spheroids and showed the superior efficacy of the ADC (Supplementary Fig. 5A). We then evaluated the binding of 6F6-ADC to membrane CLDN1 of CRC cell lines with various levels of CLDN1 expression. Conjugation to MMAE affected only minimally 6F6 binding to CLDN1 compared with the naked 6F6 mAb (Supplementary Fig. 5B). By contrast, the results of the cytotoxicity assays show that the 6F6-ADC significantly decreased CRC spheroid growth in SW620 (by 32%) and Difi cell cultures (by 73%), whereas it had no effect on SW480 cells expressing low levels of CLDN1 (Fig. 5A and Supplementary Fig. 5C). In accordance with this, treatment with 6F6-ADC reduced spheroid viability by 75% and 40% in DiFi and SW620 cells, respectively whereas it was only reduced by 5% in SW480 cells (Fig. 5B and Supplementary Fig. 5D). Corollary, propidium iodide labeling showed high cell mortality in SW620 and Difi cells expressing CLDN1 while there was no difference in mortality in the SW480 (Fig. 5C Supplementary Fig. 5E). To evaluate the therapeutic potential of 6F6-ADC, we xenografted athymic nude mice with SW620 cells and treated them with 0.9% NaCl (control-vehicle), Control-ADC, or 6F6-ADC (5 mg/kg twice per week). Tumor volume measurement over time showed that tumor growth was significantly reduced in the 6F6-ADC group compared with the control-vehicle (p < 0.001) and control-ADC (p = 0.004) groups (Fig. 5D). These results show that 6F6-ADC is specific and can efficiently reduce tumor growth of CLDN1-positive CRC cells, and that its efficacy is positively correlated with CLDN1 membrane expression.

### Combination of oxaliplatin and CLDN1-ADC: “one-two punch” therapeutic approach

Next, we combined oxaliplatin and CLDN1-ADC based on the “one-two punch” approach [20]. Twenty-four hours after seeding, we sequentially incubated SW620 cells with oxaliplatin and 72 h later with 6F6-ADC or Control-ADC for one week. This approach led to a synergistic effect of the combination at low concentrations of oxaliplatin (0.3 and 0.4 µg/ml) and 6F6-ADC (3 µg/ml) (Fig. 5E). At these concentrations oxaliplatin alone killed 32% of cells and 6F6-ADC had no effect whereas their sequential combination killed 66% of the cells. We did not observe any synergy with control-ADC. We obtained similar results with 5-FU and SN38 (active irinotecan metabolite) (Supplementary Fig. 5F). To evaluate the therapeutic potential of the sequential combination of oxaliplatin and 6F6-ADC in vivo, we subcutaneously grafted SW620 cells in mice and divided them in three groups (n = 10): (1) Untreated (Nacl), (2) oxaliplatin alone (3 mg/kg) once per week and (3) oxaliplatin (3 mg/kg) followed by 6F6-ADC (5 mg/kg; intravenous injection) 3 and 6 days later. We indeed used a suboptimal dose of oxaliplatin (3 mg/kg) on purpose, as we knew that such a concentration did not have a significant effect on tumor growth in our model but was still inducing the overexpression of membrane CLDN1 as shown in Fig. 2F. This protocol was repeated for 4 weeks. Tumor volume measurement over time showed that tumor growth was significantly reduced (p < 0.001) in the oxaliplatin + 6F6-ADC group (Fig. 5F). Moreover, the adapted Kaplan-Meier survival curve showed that the median time to reach a tumor volume of 1500 mm^3^ was 13 days longer in the oxaliplatin + 6F6-ADC group than in the oxaliplatin alone group or the untrated group (log rank p = 0.0004) (Fig. 5G). These findings indicate that the sequential combination of oxaliplatin and 6F6-ADC has a stronger therapeutic effect on tumor growth and survival than oxaliplatin alone.

## Discussion

Drug resistance in CRC is still an obstacle to an effective therapeutic outcome. More information on the molecular mechanisms underlying the emergence of resistant cells are needed to develop new therapeutic interventions. In this study, we demonstrated that the tight junction protein CLDN1 can be used as a biomarker of resistance to chemotherapy and may play an active role in its establishment, confirming its status of potential therapeutic target to circumvent resistance.

Evaluation of the histological response to chemotherapy in liver metastases from 58 patients with CRC revealed that CLDN1 overexpression was correlated with the histological minor response in non-responder patients. As the histological tumor regression is associated with the clinical outcome [21], high post-chemotherapy CLDN1 expression might indicate poor prognosis. CLDN1 upregulation following neoadjuvant chemotherapy was previously observed in patients with invasive breast cancer [40]. Similarly, high CLDN7 expression has been associated with shorter progression-free survival and poor sensitivity to platinum-based chemotherapy in patients with ovarian carcinoma [41].

We then investigated the mechanism leading to CLDN1 overexpression after chemotherapy. The starting point is the activation of p38 MAPK signaling that transduces stress signals from the environment [32]. Moreover, we previously showed that 5-FU and oxaliplatin induce p38 phosphorylation in HCT116 cells and that p38 phosphorylation level is linked to impaired response to FOLFIRI therapy in patients with CRC [33]. One of the consequences of p38 phosphorylation will be the activation of the Wnt/β-catenin signaling through the interaction of p38 with GSK3β, inducing GSK3β phosphorylation at Ser9, leading to its inactivation. It has been suggested that in thymocytes, phosphorylation by p38 is an alternative pathway for GSK3β inactivation [35]. Such a crosstalk between the p38 and the WNT pathway will lead to transcriptional activation of *CLDN1*, which is regulated by β-catenin/TCF signaling [37, 42]. This was confirmed by the abrogation of CLDN1 overexpression following treatment with chemotherapy in cells incubated with inhibitors of p38 and Wnt pathway and by functional inhibition of GSK3β. Together, these findings indicate that chemotherapy-mediated overexpression of CLDN1 is due, in part, to increased Wnt/β-catenin signaling initiated by p38 activation, thus confirming CLDN1 critical role in the regulation of Wnt/β-catenin pathway [43]. However, we cannot rule out that GSK3β could be also involved in CLDN1 upregulation by another mechanism as it was reported to regulate multiple signaling pathways, such as Sonic Hedgehog, Notch, or the Akt/mTor signaling [44, 45].

The elevated CLDN1 expression in two oxaliplatin-resistant cell lines we established [23] suggested a link between CLDN1 and chemoresistance. *CLDN1* silencing increased their sensitivity to oxaliplatin, indicating that CLDN1 overexpression enhances drug resistance and highlighting its role in the acquired chemoresistance of CRC cells. In lung cancer, CLDN1 was also shown to be involved in the development of chemoresistance to anticancer agents, including cisplatin, doxorubicin, SN-38 and gemcitabine [16]. Moreover, it promotes cisplatin resistance by activating autophagy through upregulation of ULK1 phosphorylation [46]. CLDN1 regulation of cell autophagy was also described in 5-FU resistant HepG2 (liver cancer) cells [47].

As acquired resistance to chemotherapy is a dynamic and multifactorial process, we performed RNA sequencing and GSEA to identify what pathways affected by oxaliplatin treatment were dependent on CLDN1 expression. Among the five most significant hallmarks, three pathways (inflammatory response, epithelial-to-mesenchymal transition, and KRAS signaling) were enriched in both cell lines as a signature of oxaliplatin effects. Conversely, oxidative phosphorylation and glycolysis, were decreased in control cells (shLUC). This supports the hypothesis that CLDN1 is negatively correlated with oxidative metabolic phenotypes, as previously described [48]. Genes mediating apoptosis were enriched and upregulated in CLDN1-silenced cells, such as *TXNIP* that encodes thioredoxin binding protein with proapoptotic function through the activation of apoptosis signal regulating kinase 1 (ASK1) [49], and *GADD45* that encodes a stress sensor with an important role in apoptosis induction [50]. CLDN1 anti-apoptotic function was confirmed in vitro by the increase of apoptosis in oxaliplatin-resistant CRC cell lines upon *CLDN1* silencing. To the best of our knowledge, CLDN1 implication in apoptosis was only described in transgenic mice that overexpress CLDN1 in the intestine [51] and as the causal role of resistance to anoikis in CRC cells [52]. CLDN1 role in apoptosis in other cancer types remains controversial because both anti-apoptotic [53] and pro-apoptotic roles [54] were described in breast cancer cell lines.

Taken together, these results indicate that CLDN1 could be used as a therapeutic target to circumvent drug resistance in CRC. To this end, we have previously developed an anti-CLDN1 mAb (6F6) against CLDN1 and confirmed its efficacy at inhibiting the growth of CRC cells [19]. Using an ADC approach (i.e. conjugation with MMAE), we increased the 6F6 therapeutic efficiency (cytotoxic activity). We further used a sequential combination of oxaliplatin and the 6F6-ADC in vitro and in vivo and observed a drastic synergistic effect at low concentrations of each compound. In mice xenografted with SW620 cells, this sequential combination greatly improved the therapeutic effect on tumor growth and survival compared with oxaliplatin alone. In this “one-two punch” approach, first oxaliplatin targets the bulk of tumor cells and then 6F6-ADC recognizes the residual resistant CLDN1-positive CRC cells. This strategy takes advantage of the tumor cell changes (e.g. induction of CLDN1 expression by oxaliplatin treatment) that occur during drug resistance development [55]. It is well known that chemotherapy effectiveness is limited by the selection of drug-resistant clones. Failure to eliminate these resistant cells can lead to tumor recurrence [56]. This “one-two punch” approach was first used to treat chemotherapy-induced (first punch) senescent cancer cells by senotherapy (second punch) [20] and has been tested more recently in patients with refractory breast cancer [57] and T-cell malignancies [58].

## Conclusions

To the best of our knowledge, our study showed for the first time, a high CLDN1 expression in residual chemo-resistant cancer cells in patients with metastatic CRC, thus confirming its potential use as a therapeutic target. We further showed the benefit of a sequential combination of a chemotherapeutic agent such as oxaliplatin with an anti-CLDN1-ADC based on the “one-two punch” strategy to treat patients with chemo-resistant cancer. This combination might be improved by testing, for example, FOLFOX with an ADC based on an anti-CLDN1 mAb conjugated to Exatecan (a structural analog of camptothecin) [59]. Besides providing new therapeutic opportunity to circumvent resistance and to improve the dismal outcome of patients with advanced CRC, our findings also contribute to a better understanding of the mechanisms of acquired resistance to chemotherapies used in CRC.

## Electronic supplementary material

Below is the link to the electronic supplementary material.

Supplementary Methods

Supplementary Tables

Supplementary Figures
