# Supplementary material for: Improving the response to oxaliplatin by targeting chemotherapy-induced CLDN1 in resistant metastatic colorectal cancer cells
Source: Cell Biosci. 2023 Apr 11;13:72. doi: 10.1186/s13578-023-01015-5 (PMC10091849; doi:10.1186/s13578-023-01015-5)

**Additional File 1**

**Supplementary methods**

**Proteome array**

Protein extracts were performed on SW620 cells untreated or treated with oxaliplatin at 1.2µM for 18h. Detection of activated kinases was performed using the Proteome Profiler Human Phospho-MAPK array kit (R&D systems) according to the supplier's instructions.

**Generation of ADC**

*ADC construction* All solvents were anhydrous reagents from commercial sources. Unless otherwise noted, all chemicals and reagents were obtained commercially and used without purification. TFA salt of MMAE was purchased from Levena Biopharma (#T1004). Thin layer chromatography (TLC) was performed using commercial pre-coated aluminium sheets silica gel (60 Å, F254; Merk) and revealed under UV 254 lighting. Column chromatography was carried out on an ISCO purification unit, Combi Flash RF 75 PSI, with Redisep flash silica gel columns (60 Å, 230–400 mesh, grade 9385). NMR spectra were measured on a Bruker Ultrashield 300 spectrometer, 300 MHz (1H) and 75 MHz (13C). Chemical shifts are reported in parts per million (ppm, δ), and are referenced to the residual solvent. Coupling constants are reported in hertz (Hz). The following abbreviations are used singularly or in combination to indicate the multiplicity of signals: s singlet, d doublet, t triplet, q quartet, m multiplet. The purity was determined by high performance liquid chromatography (HPLC). Purity of all final compounds was 95% or higher. HPLC analyses were carried out with a LaChrom Elite system [Hitachi L-2130 (pump), L-2200 (autosampler) and L-2400 (UV-detector)] using 254 nm UV for detection. The column was a XBridge C-18 (250 × 10 mm, 4μ, 135A); elution was performed with 0.1% (by volume) of TFA in water (solvent A), and 0.1% of TFA in acetonitrile (solvent B); gradient 20-100% of B over 35 minutes with a flow rate of 1 mL.min-1; column temperature of 25 °C; injection of 10 µL in DMSO. Semi-preparative HPLC was carried out on a Gilson PLC 2050 system ARMEN V2 (pump) equipped with ECOM TOYDAD600 (UV) for UV detection at 254 nm at 25 °C; a Waters XBridge™ C-18; 5 µm (250 mm x 19.00 mm) column was used; compounds were eluted with 0.1 % trifluoroacetic acid in water (solvent A), and acetonitrile (solvent B); as a gradient from 20 to 100% B over 32 min then 100% B for 6 min at 17.1 mL/min. High-resolution accurate mass measurements (HRAM) were performed in positive mode with an ESI source on a Q-TOF mass spectrometer with an accuracy tolerance of 2 ppm by the “Fédération de Recherche” ICOA/ CBM (FR2708) platform. Mass spectrometric analyses of ADCs were performed on a Bruker maXis mass spectrometer coupled to a Dionex Ultimate 3000 RSLC system. Prior to MS analysis, samples (ca. 5 μg) were desalted on a MassPREP desalting cartridge (2.1 × 10 mm, Waters) heated at 80 °C using 0.1% formic acid as solvent A and 0.1% formic acid in acetonitrile as solvent B at 500 μL min−1. After 1 min, a linear gradient from 5 to 90% B in 1.5 min was applied; the first 1.5 min were diverted to waste. HRMS data were acquired in positive mode with ESI source over the m/z range from 900 up to 5000 at 1 Hz and processed using DataAnalysis 4.4 software (Bruker) and MaxEnt algorithm for spectral deconvolution.

*Synthesis of linkers* Syntheses of both intermediates and final linker-drugs **diSPh-PEG_12_-VC-MMAE** were carried out according to previously described methodes [1].

*General bioconjugation procedure* BBS buffer preparation (25 mM NaCl, 1mM EDTA, pH 8.0): 27.7 mL of a 19.07 g/L concentrated solution of Borax (Na_2_B_4_O_7_.10H_2_O) in deionised water were mixed to 22.3 mL of 0.1 M HCl. Then, 73.15 mg NaCl and 18.6 mg EDTA were added. pH was adjusted to 8.0 using 0.1 M HCl. Finally, BBS buffer was conserved at 4°C. General bioconjugation protocol: TCEP (tris(2-carboxyethyl) phosphine) was dissolved in BBS buffer until 1mM concentration was reached. Antibody solutions were prepared in BBS buffer at 4.5-5.5 mg/mL concentration. Linkers were solubilised in DMSO at 1 mM concentration and conserved at 4°C.

- Bioconjugation of **diSPh-PEG_12_-VC-MMAE** linker

In 200 µL of antibody solution were added 6 eq. of TCEP (1 mM), placed at 37 °C with no stirring. Then, 6 eq. of **diSPh-PEG_12_-VC-MMAE** linker were added and the resulting solution was stirred 16h at 4°C.

*ADC purifications and sterilisations* Crude ADC solutions were purified by vivaspin (Sartorius Vivaspin 500, 10,000 MWCO, PES, 100pc) through centrifugation (5.000 G, 10 min/cycle, repeated 4 times). BBS buffer was replaced by PBS 7.2 buffer (Thermofisher Gibco™). Obtained solutions were filtrated under sterile atmosphere through a 0.22 µm filter. Then, sterile ADC solutions were freeze using liquid nitrogen and store at -80°C.

*Deconvoluted mass spectrometry of ADCs (RTX/6F6);* Mass spectrometric analyses of ADCs were performed on a Bruker maXis mass spectrometer coupled to a Dionex Ultimate 3000 RSLC system. Prior to MS analysis, samples (ca. 5 μg) were desalted on a MassPREP desalting cartridge (2.1 x 10 mm, Waters) heated at 80 °C using 0.1% formic acid as solvent A and 0.1% formic acid in acetonitrile as solvent B at 500 μL/min. After 1 min, a linear gradient from 5 to 90% B in 1.5 min was applied; the first 1.5 min were diverted to waste. MS data were acquired in positive mode with ESI source over the m/z range from 900 up to 5000 at 1 Hz. Data were processed using DataAnalysis 4.4 software (Bruker) and MaxEnt algorithm for spectral deconvolution. Deconvolution was carried out in the range 20-180 kDa, with results recorded for full antibody as well as fragments comprising L, LH, HH, LHH as a result of antibody dissociation within the MS. Not all species were observed for all ADCs. DAR was approximated for each species as an average of the percentage abundance of each DAR species present, with the quantities calculated by peak integration of the first glycosylation peak, following the formula:

$${DAR}_{average}= \frac{{DAR}_{LHHL}+\left( {DAR}_{LHH}+ {DAR}_{L} \right)+2*{DAR}_{LH}}{3}$$

[1] F. Bryden, C. Martin, S. Letast, E. Lles, I. Viéitez-Villemin, A. Rousseau, C. Colas, M. Brachet-Botineau, E. Allard-Vannier, C. Larbouret, M.C. Viaud-Massuard, N. Joubert, Impact of cathepsin B-sensitive triggers and hydrophilic linkers on in vitro efficacy of novel site-specific antibody-drug conjugates, Org. Biomol. Chem. 16 (2018) 1882–1889. doi:10.1039/c7ob02780j.

**ADC 6F6 PEG_12_ VC MMAE** with DAR_average_= 3.49


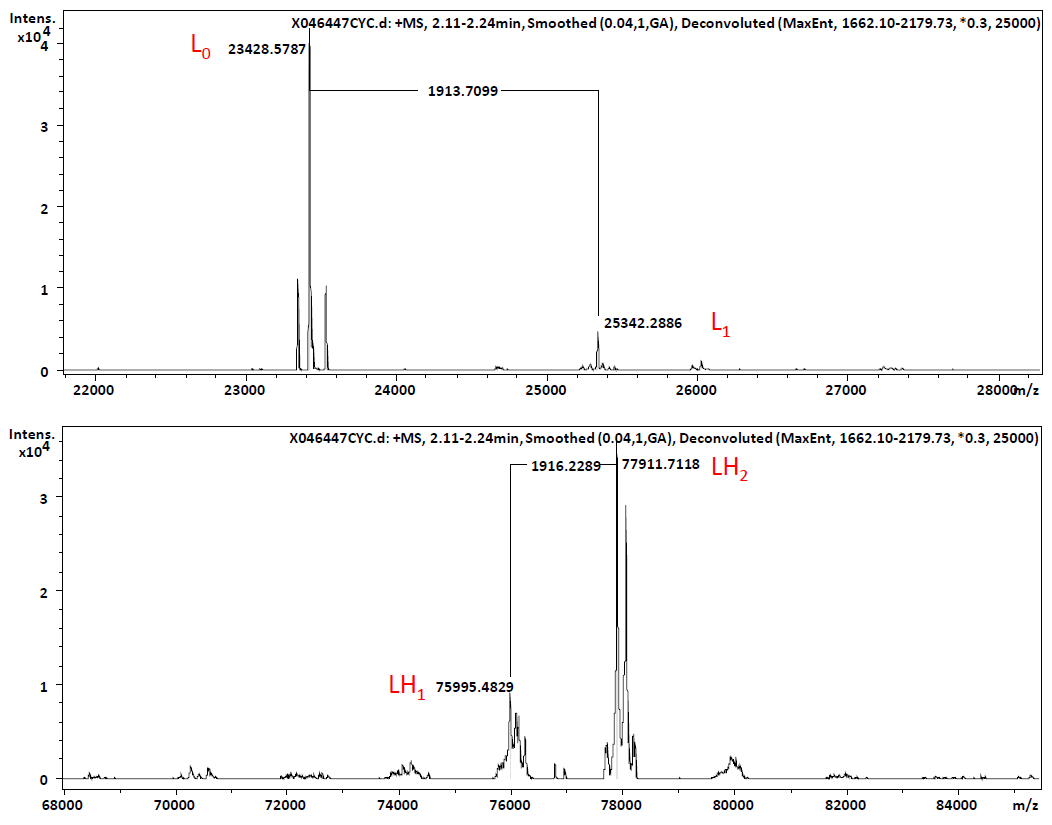


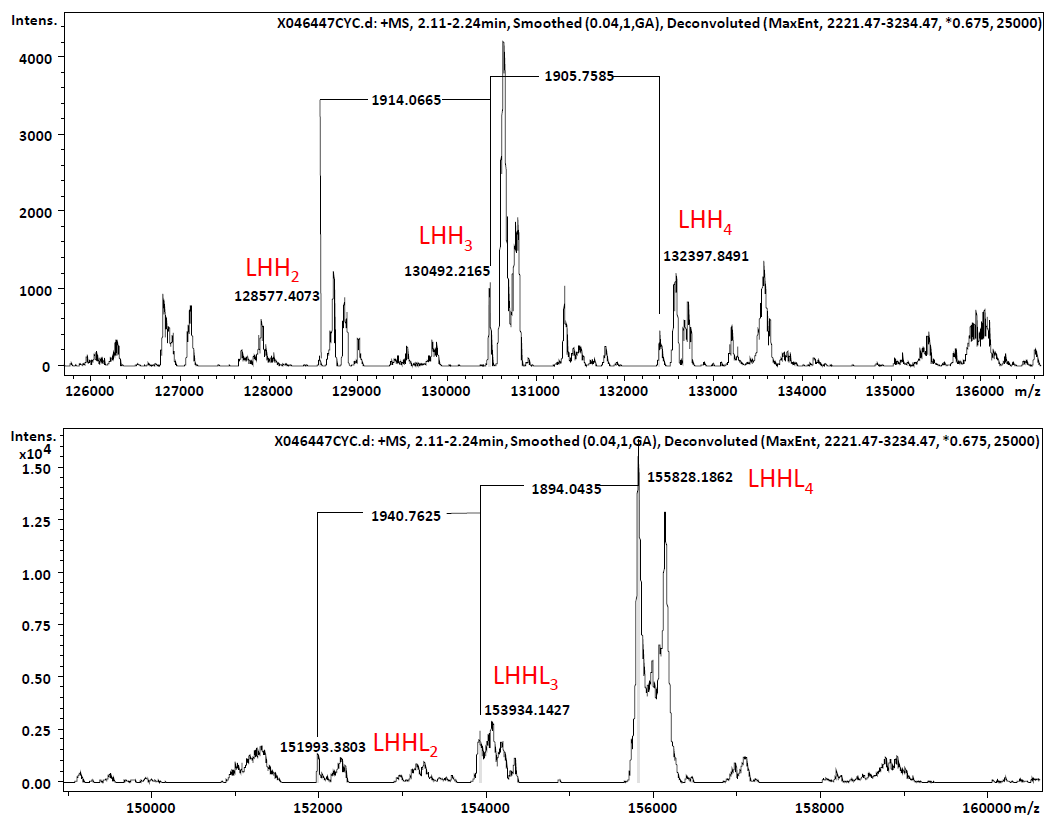

**ADC CTRL PEG_12_ VC MMAE** with DAR_average_= 3.70


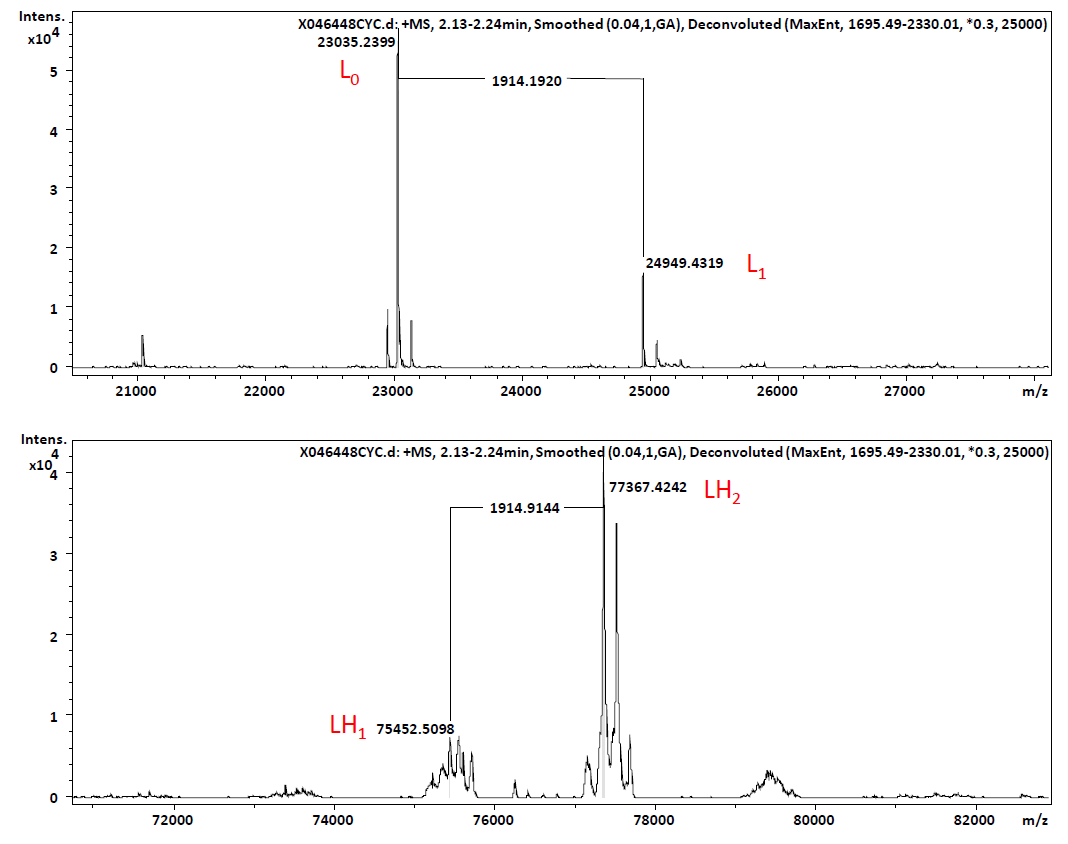


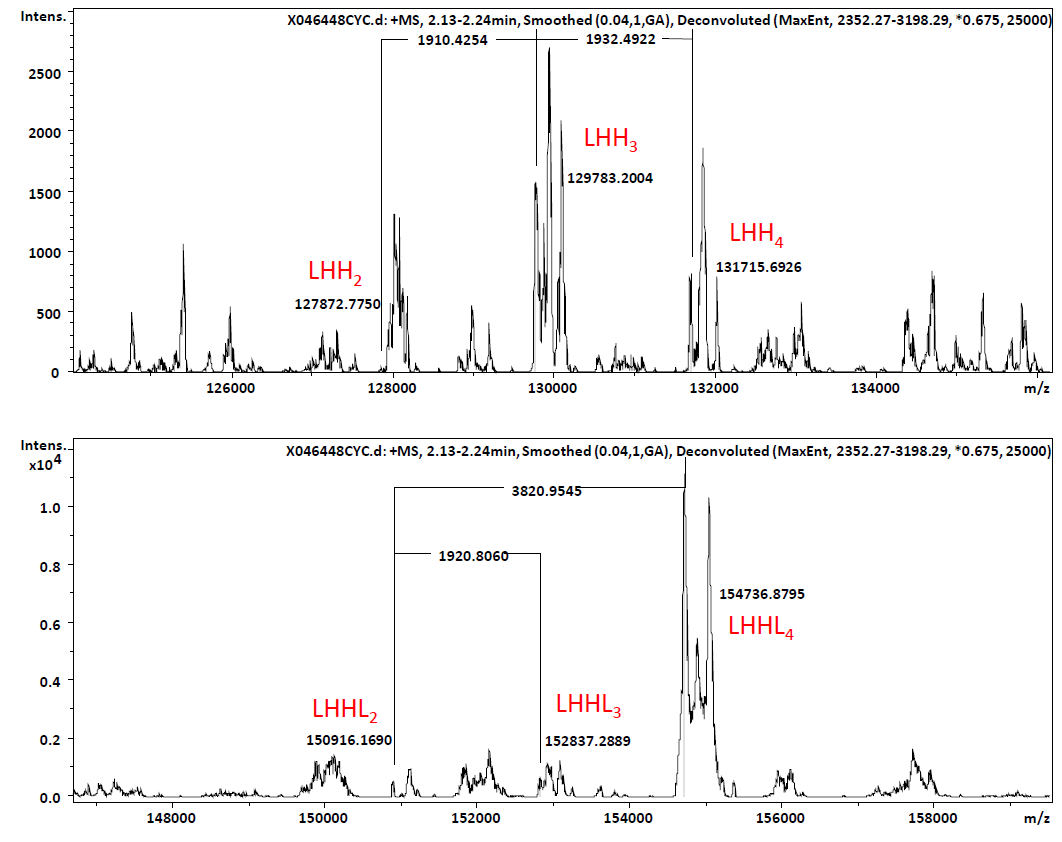

Supplement: Supplementary file 1 — Supplementary Methods [file 13578_2023_1015_MOESM1_ESM.docx]
