# Supplementary material for: Improving the response to oxaliplatin by targeting chemotherapy-induced CLDN1 in resistant metastatic colorectal cancer cells
Source: Cell Biosci. 2023 Apr 11;13:72. doi: 10.1186/s13578-023-01015-5 (PMC10091849; doi:10.1186/s13578-023-01015-5)
Supplement: Supplementary file 2 — Supplementary Tables [file 13578_2023_1015_MOESM2_ESM.docx]

**Additional file 2**

**Supplementary tables**

Supplementary table 1 : GSEA analysis: additional enriched hallmarks modules.

Supplementary table 2. Primer sequences

.

| **shRNA target sequences.** |
| --- |
| **shCLDN1** 5' GCAATAGAATCGTTCAAGA 3' |
| **shLUC:** 5’ TTACGCTGAGTACTTCGA 3’. |
| **shGSK3**: 5' CCCAAACTACACAGAATTTAA 3' |
| **PCR primer sequences** |
| **Gene Symbol Forward Sequence Reverse Sequence** |
| **CLDN1** 5’ TTGGGCTTCATTCTCGCCTT 3’ 5’ TTGCTTGCAATGTGCTGCTC 3’ |
| **AXIN2** 5’ GTGATGGAGGAAAATGCCTACC 3’ 5’ GTCCCCCATTACTCATGTAAGC 3’ |
| **c-MYC** 5’ TCCCTCCACTCGGAAGCACT 3’ 5’ CGGTTGTTGCTGATCTGTCTCA 3’ |
| **CCDN1** 5’ CATGGAACACCAGCTCCTCTG 3’ 5’ GTTCATGGCCAGCGGGAAGAC 3’ |
