# Supplementary material for: Improving the response to oxaliplatin by targeting chemotherapy-induced CLDN1 in resistant metastatic colorectal cancer cells
Source: Cell Biosci. 2023 Apr 11;13:72. doi: 10.1186/s13578-023-01015-5 (PMC10091849; doi:10.1186/s13578-023-01015-5)
Supplement: Supplementary file 3 — Supplementary Figures [file 13578_2023_1015_MOESM3_ESM.docx]

**Additional File 3**

**Supplementary Figures**

**B**

**A**

**Supplementary Fig. 1**. CLDN1 is overexpressed in colorectal cancer cells after incubation with oxaliplatin. **(A)** Relative *CLDN1* mRNA expression in HCT116 and Difi colorectal cancer cells incubated (+) or not (-) with 1.2 µM oxaliplatin for 72h. **(B)** Membrane CLDN1 expression analyzed by flow cytometry in the two cell lines and quantification of membrane CLDN1 expression. * p ≤0.05, ** p ≤0.01, *** p ≤0.001 (Student’s *t*-test).


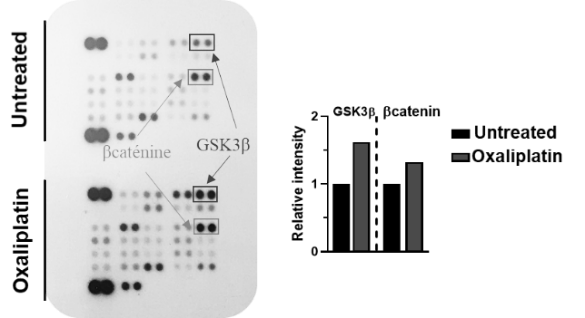

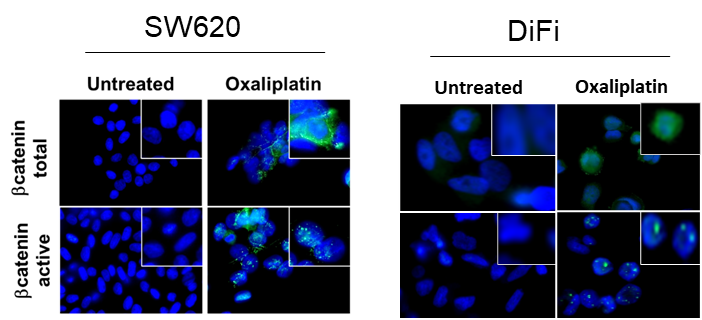

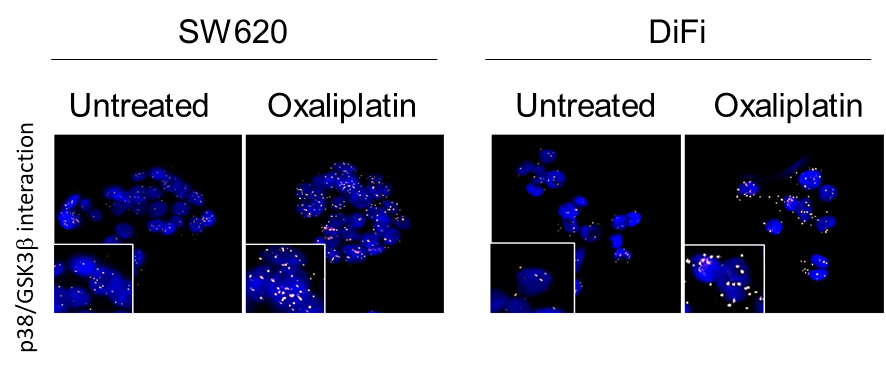

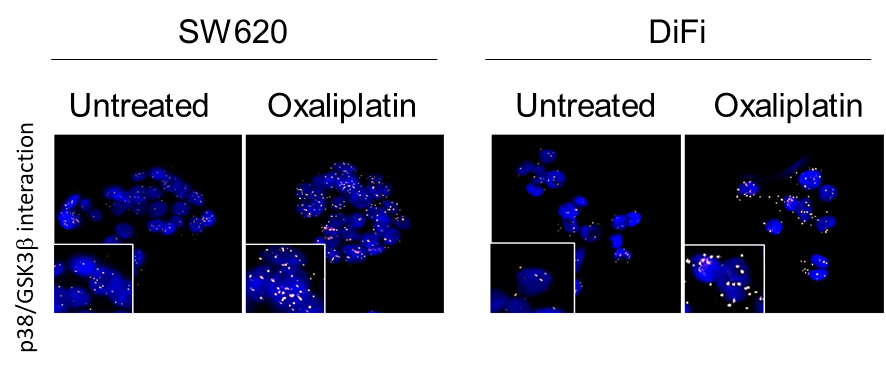


**D**

**A**

**Supplementary Fig. 2.** Oxaliplatin-mediated membrane CLDN1 overexpression is dependent on the MAPK p38,/GSK3β/Wnt-β-catenin signaling cascade**. (A)** Phospho-protein array analysis in SW620 cells after 18h incubation or not with 1.2 µM oxaliplatin. Right**,** quantification of GSK3β^Ser9^ and β-catenin expression. **(B)** Proximity ligation assay (PLA) with oligonucleotide-conjugated antibodies against p38 and GSK3β in Difi cells incubated or not with oxaliplatin (5µM for 24h). Left: Fluorescence images, nuclei were counterstained with DAPI (blue). Right: PLA dot counts per cell in the corresponding fluorescence images. **(C)** Expression and localization of total and inactive (phosphorylated) β-catenin by immunofluorescence analysis in Difi cells after incubation or not with oxaliplatin (5µM for 72h). **(D)** The TOP-/FOP Flash luciferase assay shows the transcriptional activation of the Wnt/β-catenin signaling pathway after oxaliplatin incubation (5µM for 72h) in Difi cells. **(E)** Effect of oxaliplatin (5µM for 72h) on the mRNA expression of Wnt /β-catenin target genes in Difi cells.

**E**

**C**

**B**

**B**

**E**

**Oxaliplatin-mediated membrane CLDN1 overexpression is dependent on the MAPK p38,/GSK3β/Wnt-β-catenin signaling cascade.**

**(A)** Phospho-protein array analysis in SW620 cells after 18h incubation or not with 1.2 µM oxaliplatin. Right**,** quantification of GSK3β^Ser9^ and β-catenin expression. **(B)** Proximity ligation assay (PLA) with oligonucleotide-conjugated antibodies against p38 and GSK3β in Difi cells incubated or not with oxaliplatin (5µM for 24h). Left: Fluorescence images, nuclei were counterstained with DAPI (blue). Right: PLA dot counts per cell in the corresponding fluorescence images. **(C)** Expression and localization of total and inactive (phosphorylated) β-catenin by immunofluorescence analysis in Difi cells after incubation or not with oxaliplatin (5µM for 72h). **(D)** The TOP-/FOP Flash luciferase assay shows the transcriptional activation of the Wnt/β-catenin signaling pathway after oxaliplatin incubation (5µM for 72h) in Difi cells. **(E)** Effect of oxaliplatin (5µM for 72h) on the mRNA expression of Wnt /β-catenin target genes in Difi cells.

HCT116-ROX_shCLDN1

HCT116-ROX_shLUC

Control

SW620-ROX_shCLDN1

SW620-ROX_shLUC

Control

**A**

**Supplementary Fig. 3.** *CLDN1* silencing. **(A)** Membrane CLDN1 expression determined by FACS in the oxaliplatin-resistant SW620-ROX and HCT116-ROX cell lines that express shCLDN1 or shLUC. **(B)** CLDN1 expression by western blotting in SW620_ROX-shLUC and –shCLDN1 cells.

**B**

GAPDH

CLDN1


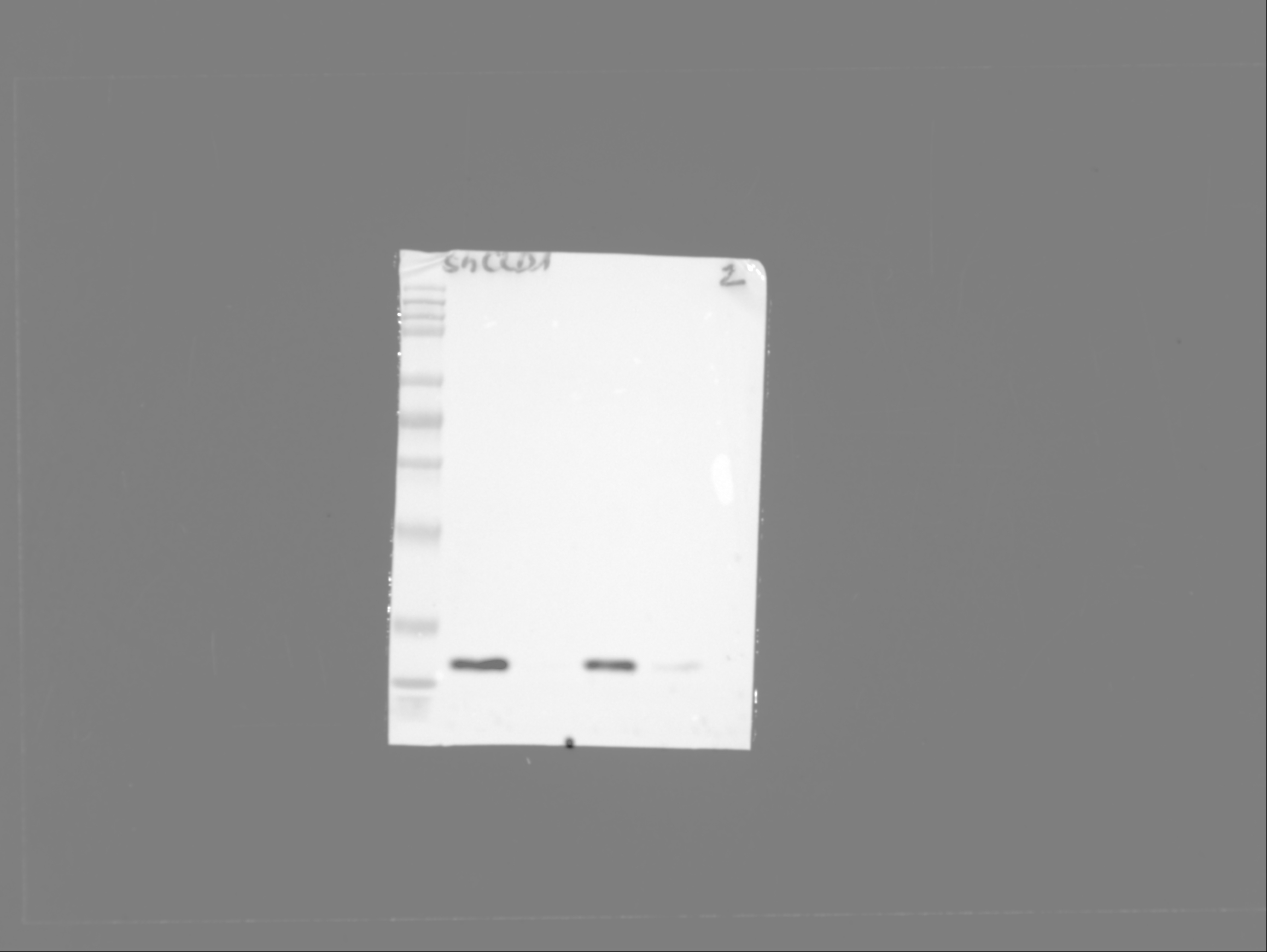

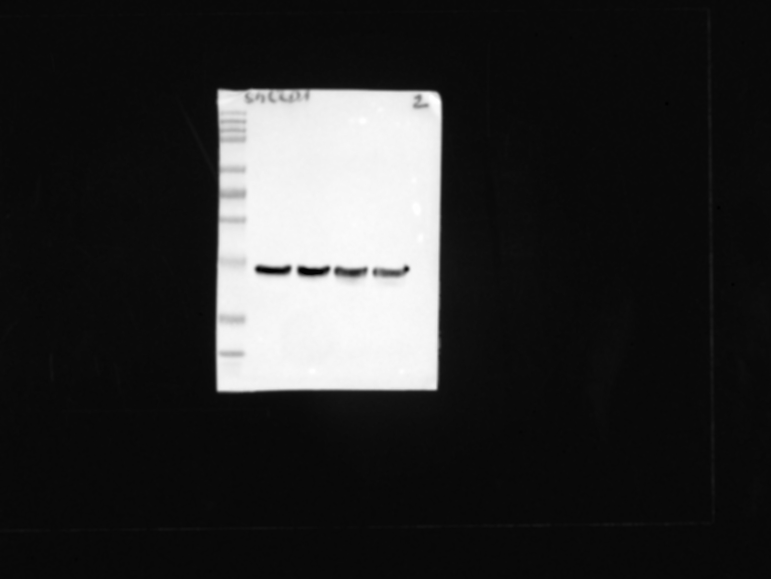


shLUC

shCLDN1

SW620-ROX


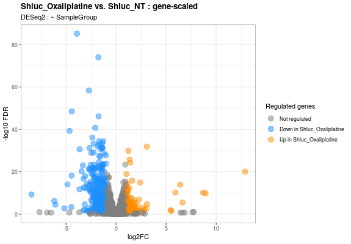

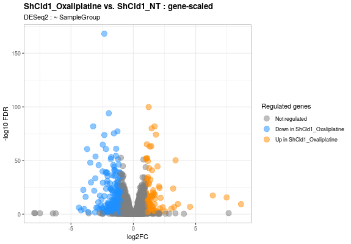

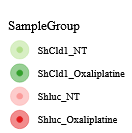

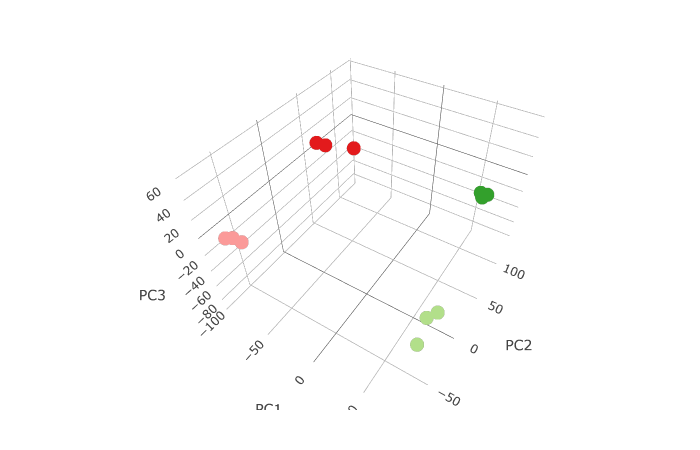

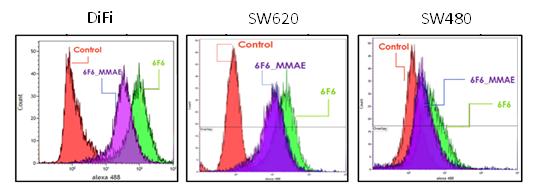

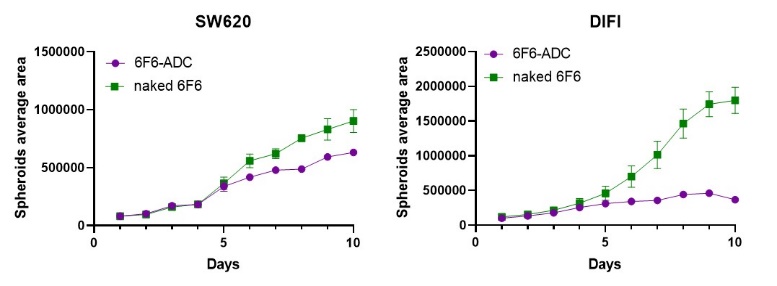


**Supplementary Fig. 4.** RNA-seq analysis of oxaliplatin-resistant SW620_ROX cells. **(A)** Principal component analysis (PCA) 3D mapping of RNA-seq data (n=3 for each cell line). **(B)** Volcano plots showing the distribution of all differentially expressed genes (blue = downregulated; orange = upregulated) after incubation with oxaliplatin according to their fold change (FC) and p value (FDR). **(C)** Enrichment plots for three dysregulated hallmarks in SW620_ROX–shCLDN1 cells compared with –shLUC cells. The location of the gene set members is indicated by vertical black lines; significant positive enrichments on the left and right indicate upregulation and downregulation of genes, respectively. **(D)** Comparison of the expression level of two pro-apoptotic genes in all analyzed samples; cpm, counts per million mapped reads.

**D**


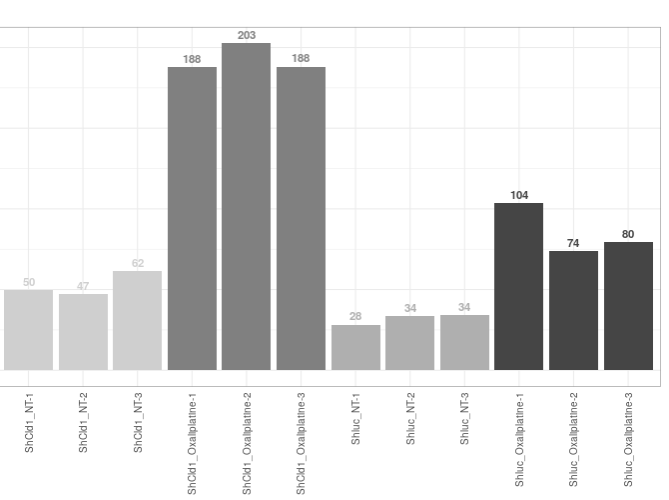

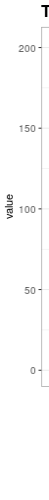

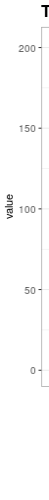

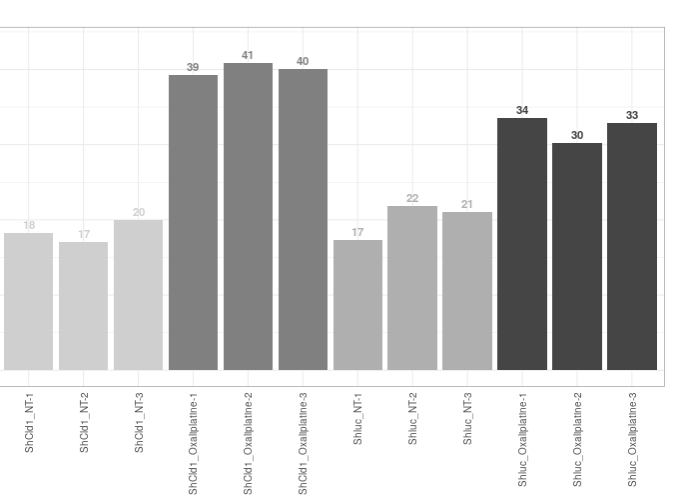


GADD45B

TXNIP


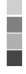


shCLDN1_NT

shCLDN1_OX

shLuc_NT

shLuc_OX

**C**


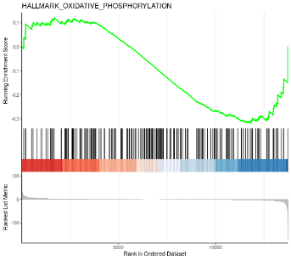

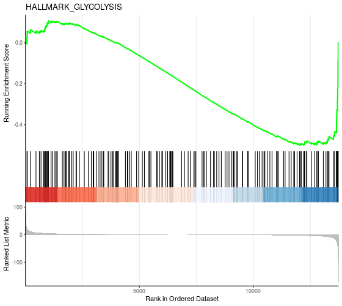

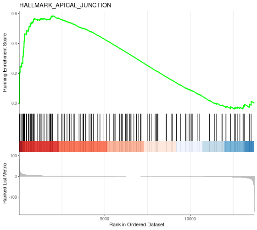

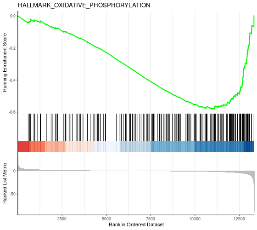

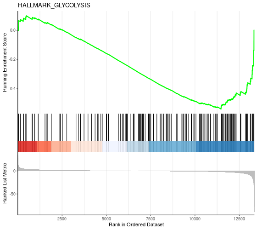

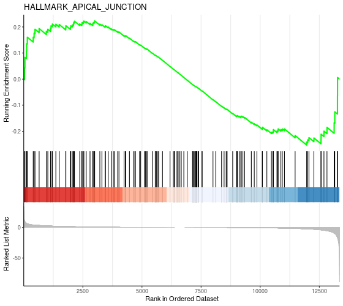


-shCLDN1

-shLUC

Oxidative phosphorylation Glycolysis Apical junction

*adjusted p = 0.0016*

*adjusted p = 0.939*

*adjusted p= 0.620*

*adjusted p =0.0068*

*adjusted p = 0.0385*

*adjusted p =0.0057*

**B**

SW620_ROX

-shCLDN1

-shLUC

**A**

**B**

**A**


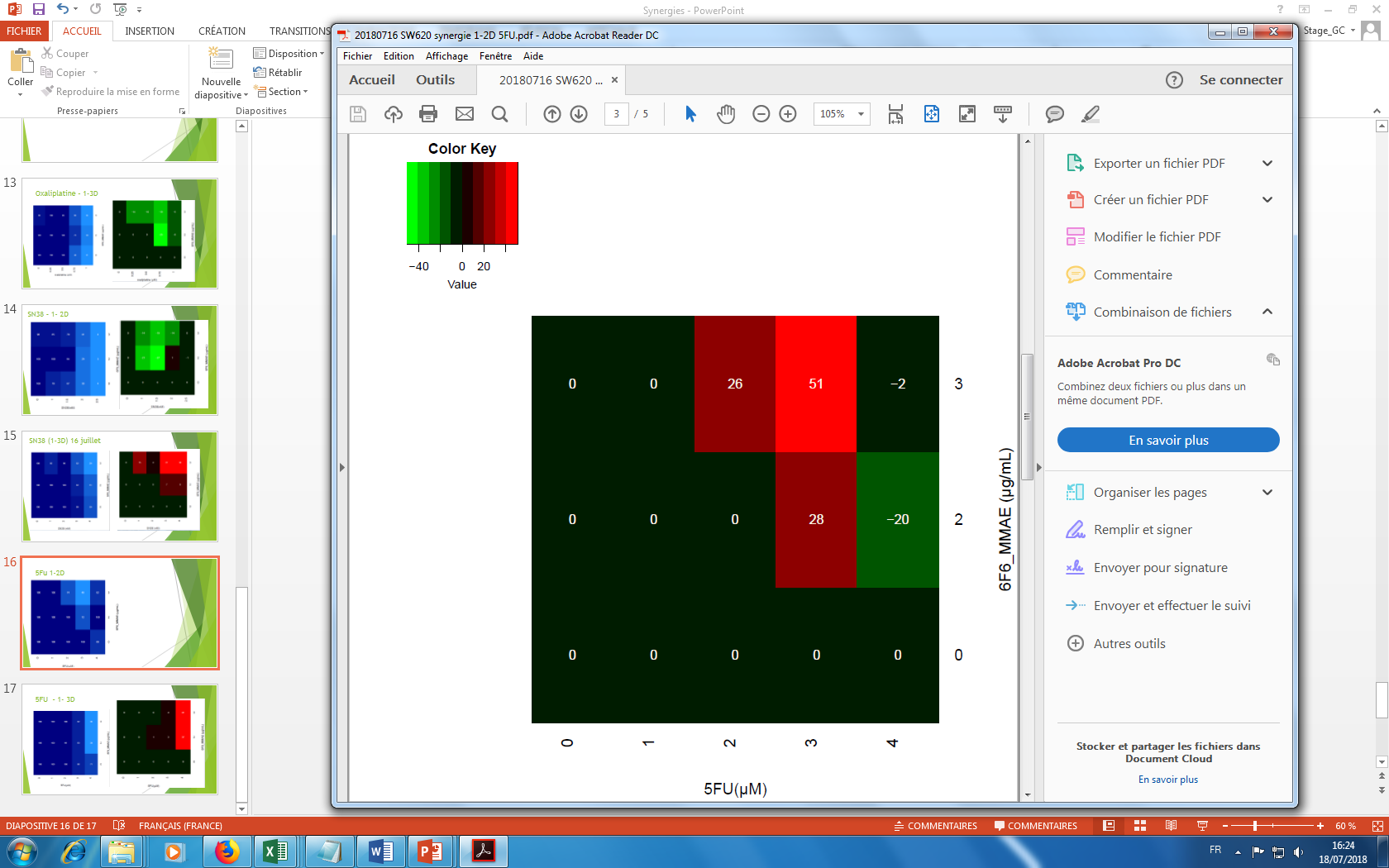

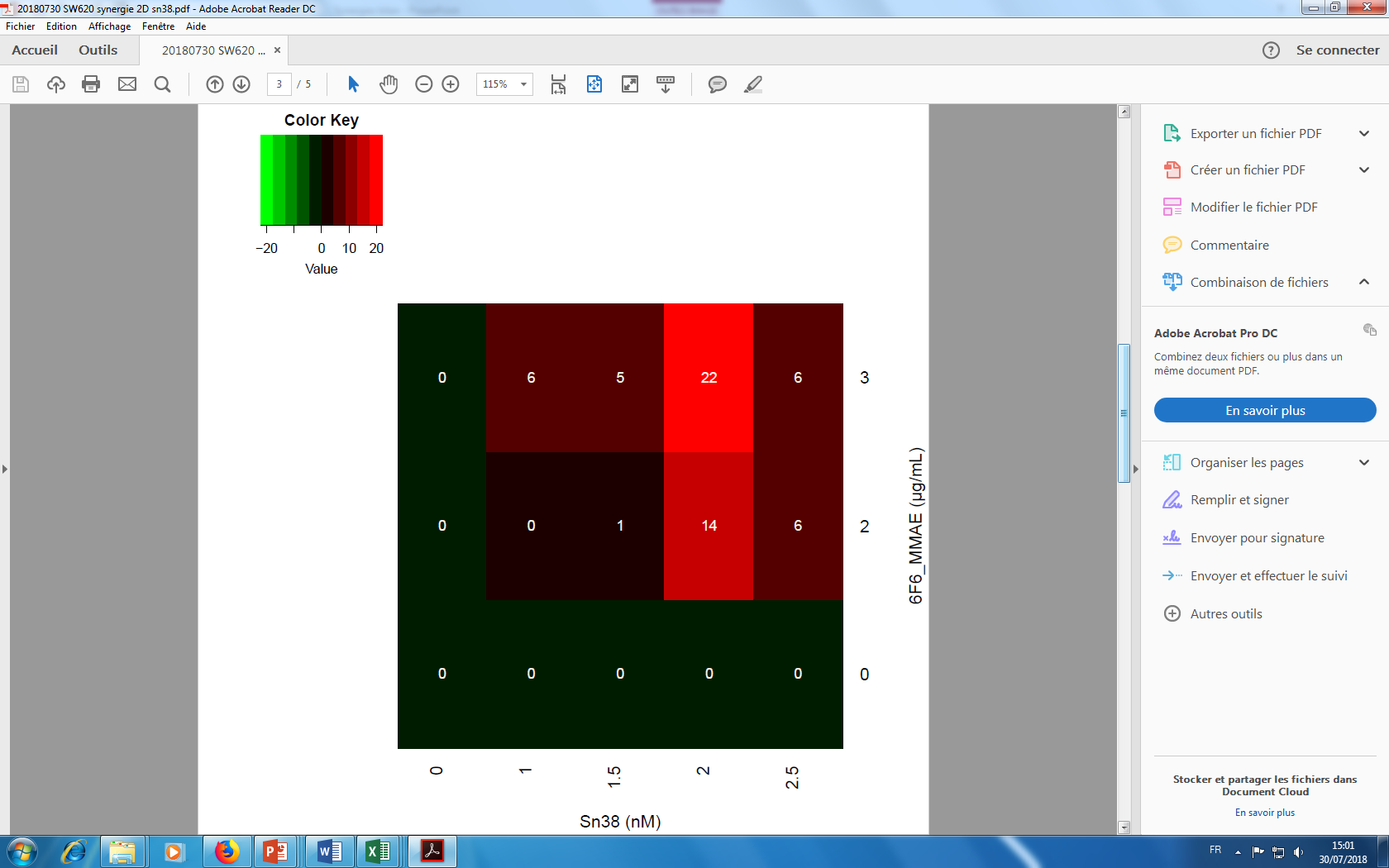

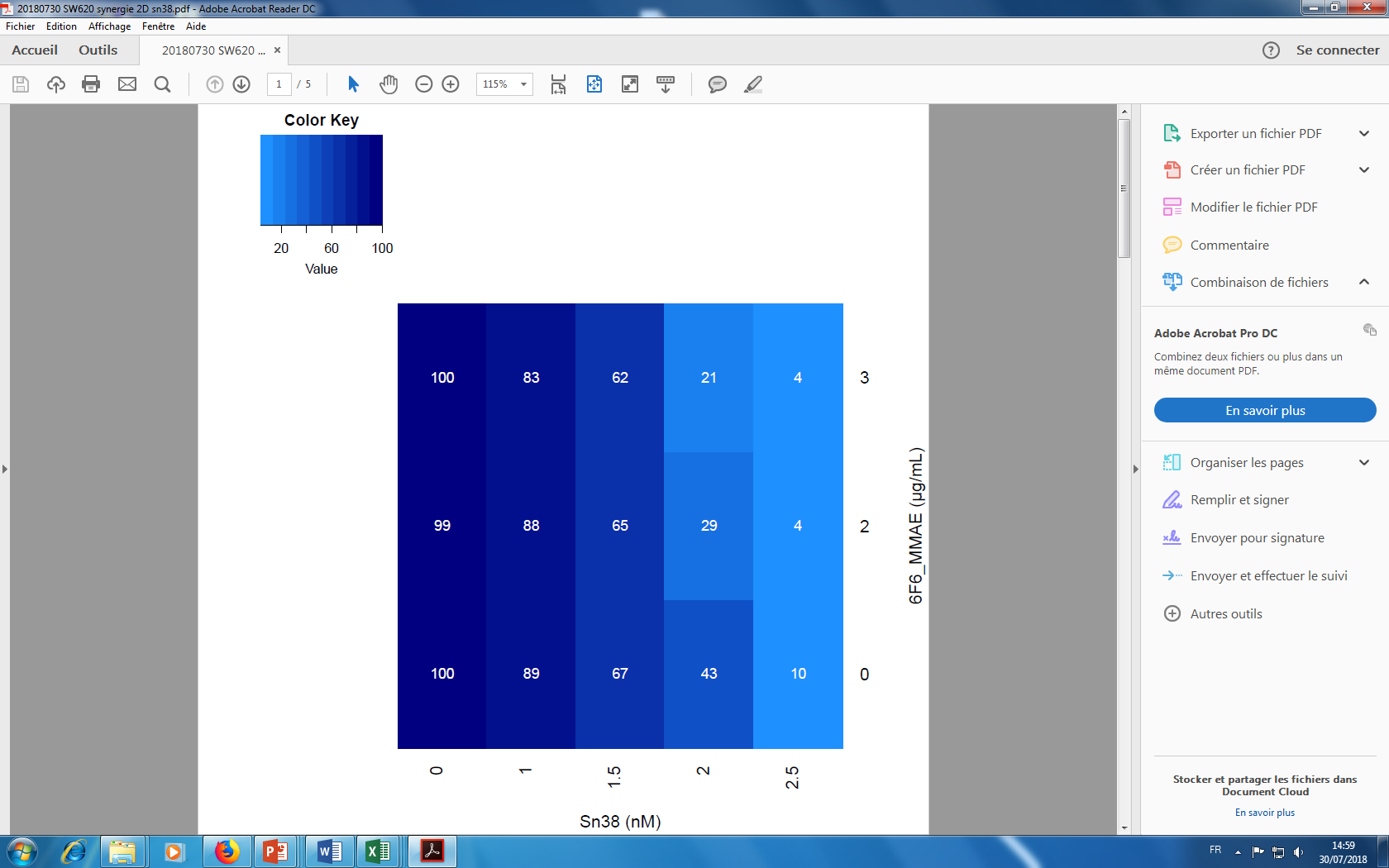

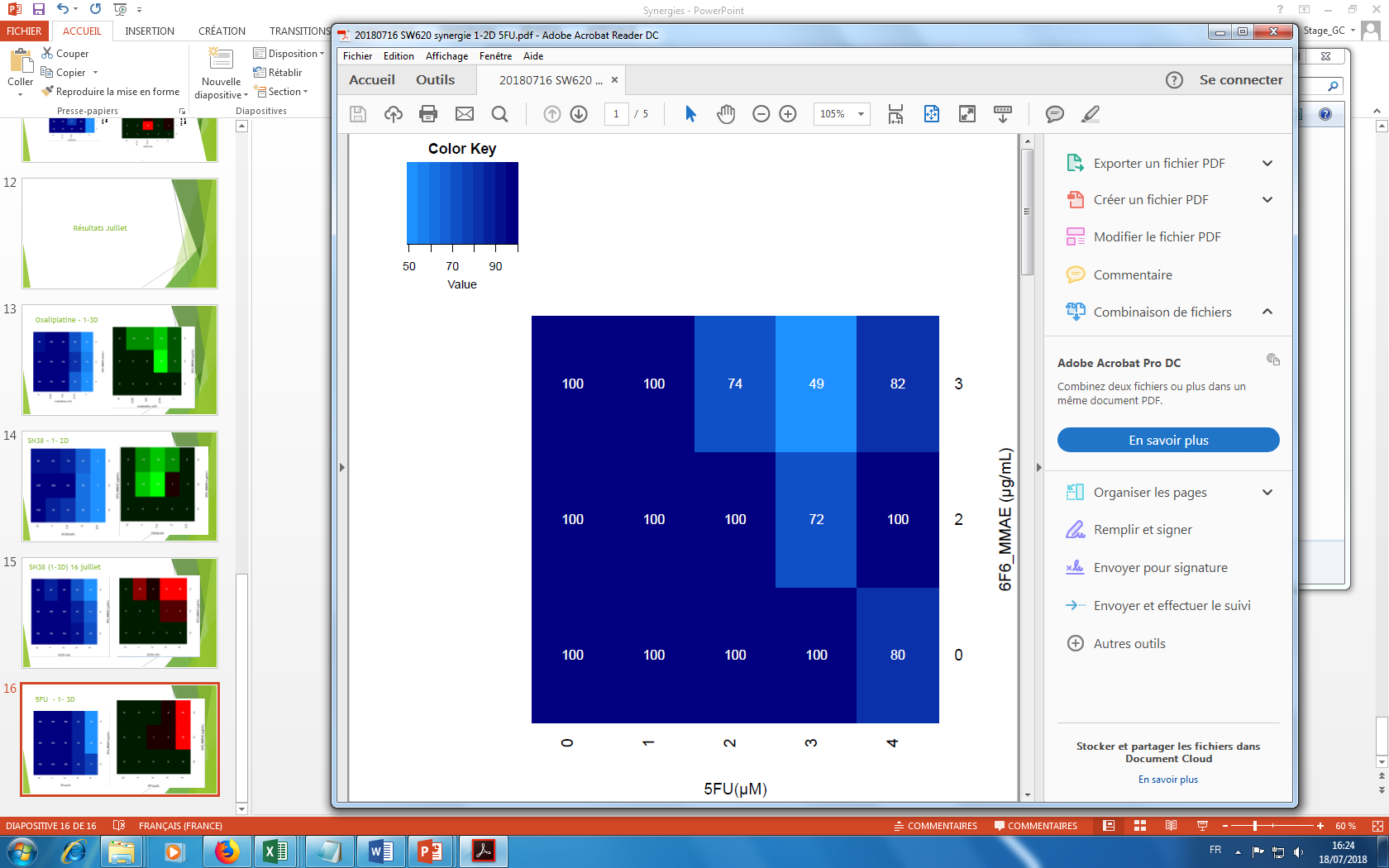


**5-FU [µM]**

**5-FU [µM]**

**Sn38 [µM]**

**Sn38 [µM]**

Synergy matrix

Survival matrix

Survival matrix

Synergy matrix

**F**

**E**

**D**

**C**

**Supplementary Fig. 5.** Therapeutic effect of an ADC anti-CLDN1 on colorectal cells **(A)** Comparison between the anti-CLDN1 mAb (6F6), naked or conjugated with MMAE (ADC) on SW620 and DIFI cell spheroids. **(B)** Comparative binding of anti-CLDN1 naked 6F6 or 6F6-ADC on three colorectal cell lines. **(C)** Growth curve of Difi or SW480 spheroids treated for 7 days with 10μg/mL of CLDN1-ADC or Control-ADC and monitored by Celigo **(D)** The cell survival at the end-point was determined by a cytotoxicity assay, the luminescence reflecting viable cells was measured. **(E)** Spheroids were incubated with 1μg/m of propidium iodide (PI) which emits a red fluorescence red when incorporated into cells. Image acquisition was performed using the Celigo™ **(F)** In vitro combination of 5-FU or SN38 (active metabolite of irinotecan) with CLDN1-ADC on the SW620 cell line. The cells were treated 24 hours after seeding with increasing doses of drug and 72 hours later with increasing doses of CLDN1-ADC. One week later a cell viability test is performed. The blue matrices represent the cell viability. The red and black matrices represent the synergy of the two molecules (red: synergy, black: additivity, green: antagonism)

CLDN1_ADC

CLDN1_ADC


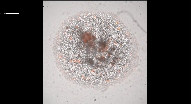

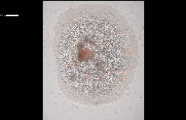

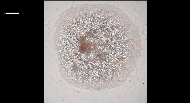

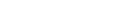

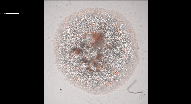

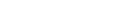


SW480


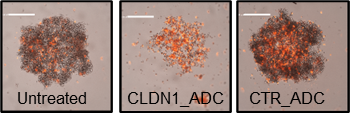


DiFi


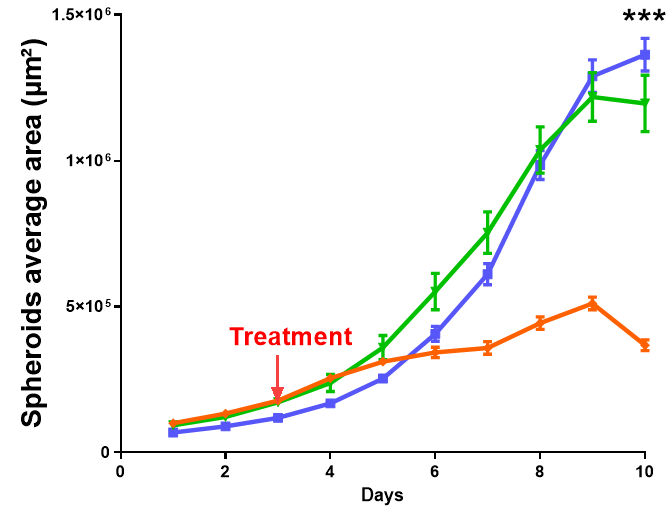


Difi

SW480

Untreated

CTR_ADC

CLDN1_ADC


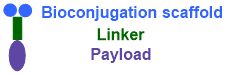

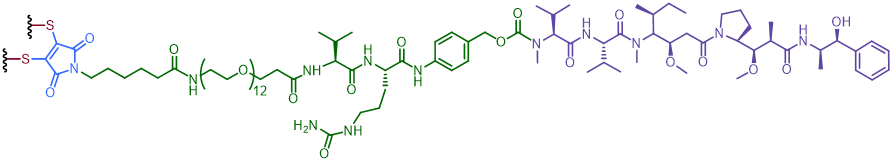


**C**

**B**

**A**

**Supplementary Fig. 6.** Schematic representations of antibody-drug conjugates (ADCs). **(A)** Schematic representations of ADCs, generated from the site-specific bioconjugation of our cleavable linkers onto mildly reduced interchain disulfide bridges of IgG1 antibodies (6F6 or control), where n is the drug-to-antibody ratio (DAR). **(B)** Chemical structure of a next generation maleimide (NGM) used as the trifunctional bioconjugation scaffold of our linker, simultaneously able to rebridge two cysteines resulting from the mild reduction of interchain disulfide bridges of IgG1 antibodies, while grafting one cytotoxic agent per disulfide bridge. **(C)** Structure of a cleavable linker, including a NGM, a polyethyleneglycol (PEG) spacer unit, a valine-citrulline-paraaminobenzyl-carbamate (VC-PABC) trigger sensitive to cathepsin B, and the cytotoxic compound monomethyl auristatin E (MMAE).

MMAE

NGM

VC-PABC

MMAE

Cathepsin B-sensitive

cleavable linker

next generation maleimide (NGM)

**ADC**
